# The IKAROS Interaction with a Complex Including Chromatin Remodeling and Transcription Elongation Activities Is Required for Hematopoiesis

**DOI:** 10.1371/journal.pgen.1004827

**Published:** 2014-12-04

**Authors:** Stefania Bottardi, Lionel Mavoungou, Helen Pak, Salima Daou, Vincent Bourgoin, Yahia A. Lakehal, El Bachir Affar, Eric Milot

**Affiliations:** Maisonneuve Rosemont Hospital Research Center, Maisonneuve-Rosemont Hospital and Faculty of Medicine, University of Montreal, Montreal, Quebec, Canada; Cincinnati Children's Hospital Medical Center, United States of America

## Abstract

IKAROS is a critical regulator of hematopoietic cell fate and its dynamic expression pattern is required for proper hematopoiesis. In collaboration with the Nucleosome Remodeling and Deacetylase (NuRD) complex, it promotes gene repression and activation. It remains to be clarified how IKAROS can support transcription activation while being associated with the HDAC-containing complex NuRD. IKAROS also binds to the Positive-Transcription Elongation Factor b (P-TEFb) at gene promoters. Here, we demonstrate that NuRD and P-TEFb are assembled in a complex that can be recruited to specific genes by IKAROS. The expression level of IKAROS influences the recruitment of the NuRD-P-TEFb complex to gene regulatory regions and facilitates transcription elongation by transferring the Protein Phosphatase 1α (PP1α), an IKAROS-binding protein and P-TEFb activator, to CDK9. We show that an IKAROS mutant that is unable to bind PP1α cannot sustain gene expression and impedes normal differentiation of Ik^NULL^ hematopoietic progenitors. Finally, the knock-down of the NuRD subunit Mi2 reveals that the occupancy of the NuRD complex at transcribed regions of genes favors the relief of POL II promoter-proximal pausing and thereby, promotes transcription elongation.

## Author Summary

Perturbation of the expression level of IKAROS, a transcription factor critical during hematopoiesis, is associated with malignant transformation in mice and humans. The importance of IKAROS expression levels for the control of target-gene regulation was addressed in hematopoietic progenitor cells. The collaboration between IKAROS and the Nucleosome Remodeling and Deacetylase (NuRD) complex can promote gene activation or repression. IKAROS can also interact with the Positive-Transcription Elongation Factor b (P-TEFb) and the Protein Phosphatase 1 (PP1), an important P-TEFb regulator. Immunoaffinity purification of IKAROS interacting proteins and Fast Protein Liquid Chromatography analysis revealed a dynamic interaction between IKAROS, PP1 and the newly defined NuRD-P-TEFb complex. This complex can be targeted to specific genes in cells expressing high levels of IKAROS to promote productive transcription elongation. Based on our results we suggest that, in addition to P-TEFb, the NuRD complex and PP1 are required to facilitate transcription elongation of IKAROS-target genes and normal differentiation of hematopoietic progenitor cells.

## Introduction

The tumor suppressor IKAROS is a transcription factor critical for hematopoietic multi-lineage priming, cell fate and lineage determination [1]–[5]. Mice homozygote for the *Ikaros* null mutation (Ik^NULL^) display severe defects in lymphocyte development and function, and develop leukemias and lymphomas with complete penetrance [6]. These phenotypes reflect the requirement of IKAROS to activate the lymphoid program in hematopoietic progenitor cells (HPCs) [4]. IKAROS is also involved in transcriptional regulation of erythroid- and myeloid-specific genes [7]–[13]. The hematopoietic differentiation is affected not only by the presence or absence of IKAROS, but also by its relative expression level [14]. In particular, during B-cell progenitor differentiation, dynamic change of IKAROS expression level has been identified as a key regulator for the expression of multiple target genes [15], [16].

IKAROS controls chromatin organization mainly through association with the Nucleosome Remodeling and Deacetylase (NuRD) complex [5], [17], [18]. NuRD was initially identified as a repressive complex but it was demonstrated afterwards to promote transcription of specific genes as well [5], [19]–[22]. It remains to be defined how this HDAC-containing complex activates transcription. IKAROS contributes to the assembly and stability of the pre-initiation complex (PIC) at promoters [13], [23]–[26] and interacts directly with CDK9, the catalytic subunit of P-TEFb (Positive-Transcription Elongation Factor b) [27]. CDK9 phosphorylates the C-terminal domain (CTD) of the large subunit of RNA Polymerase II (POL II) on Ser2 as well as the SPT5 subunit of DSIF and the E subunit of NELF. These events are required to release promoter-proximal paused POL II and thus, allow productive transcription elongation. Most nuclear P-TEFb is sequestered in the 7SK snRNP repressive complex. This repressive complex is characterized by the snRNP molecule and the proteins HEXIM (HEXIM1 or 2), LARP7 and MePCE [28]. Of interest here, is the dissociation of the P-TEFb from this repressive complex promoted by the sequential activity of the protein phosphatase 2B (PP2B) that favors conformation changes of the 7SK snRNP and protein phosphates 1α (PP1α), involved in CDK9 dephosphorylation at different residues including Thr186 and Ser175 [29], [30]. Dephosphorylated CDK9/P-TEFb is preferentially recruited to promoters by the general factor BRD4 or specific transcription factors such as HIV TAT [31]–[33]. Then, CDK9/P-TEFb becomes catalytically active and promotes the release of promoter-proximal paused POL II when it is “re-phosphorylated” by the TFIIH associated CDK7 [30]. PP1α is one of the three catalytic subunits (α, β or γ) which, together with a regulatory subunit, forms each PP1 enzyme [34]. Interestingly, IKAROS interacts with PP1 and is dephosphorylated by this phosphatase [35]. Whether the IKAROS-PP1 interaction is important for Cdk9/P-TEFb activation and thus, transcription elongation of IKAROS-target genes is not known.

Here, we sought to define the importance of these protein associations for IKAROS and NuRD to function as transcriptional activators. We demonstrate that IKAROS is an adaptor molecule required for the recruitment of the newly identified NuRD-P-TEFb complex to IKAROS-target genes. IKAROS binding to the promoter region of specific genes is also associated with the local recruitment of the CDK9/P-TEFb activator, PP1α. Interestingly, the Mi2/NuRD occupancy at IKAROS-target genes is enhanced when transcription elongation is proficient, and the release of POL II promoter-proximal pausing is decreased in the absence of Mi2. Our data also reveal that the dynamic interaction of IKAROS with the NuRD-P-TEFb complex depends on IKAROS protein levels. Low-levels of IKAROS suffice for Mi2/NuRD recruitment to promoters, but higher levels of IKAROS are required for NuRD-P-TEFb and PP1α to chromatin, CDK9/P-TEFb activation, and productive transcription elongation. Finally, we demonstrate that IKAROS-PP1α protein interaction is required for normal hematopoietic differentiation of primary HPCs.

## Results

### IKAROS interacts with the newly characterized NuRD-P-TEFb complex

**Figure 1:**
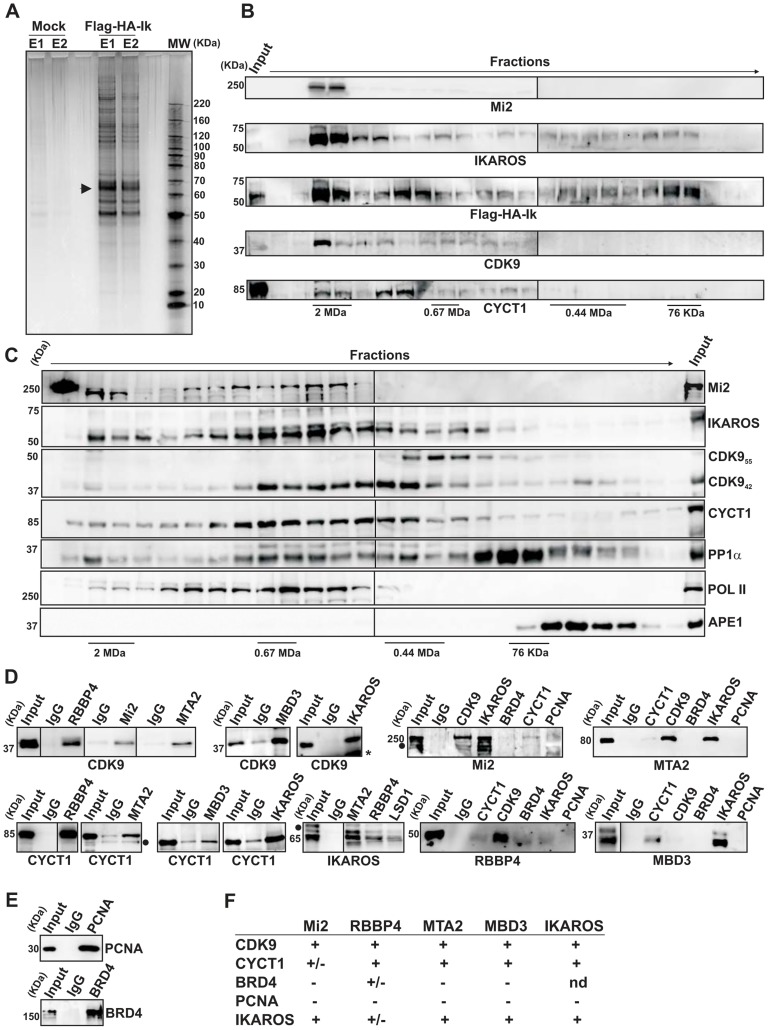
IKAROS interacts with the newly characterized NuRD-P-TEFb complex. **A**) Purification of Flag-HA-Ik associated proteins. Nuclear extracts from Jurkat cells carrying the pOZ-N-Flag-HA-IKAROS-IRES-IL2R vector and expressing a double tagged IKAROS (Flag-HA-Ik) or Jurkat cells expressing the pOZ-N-Flag-HA-IRES-IL2R empty vector (Mock) were used for sequential immunoaffinity purification using Flag- followed by HA-conjugated matrix. A fraction of the purified complexes were loaded on SDS-PAGE and silver stained; E1: first HA elution; E2: second HA elution; MW: molecular weights (in KDa); the Flag-HA-Ik protein is indicated by an arrow; **B, C**) Molecular weight fractionation of the IKAROS-associated complexes. Flag-HA-Ik immunoaffinity purified complexes (panel B) or nuclear extracts (panel C) were fractionated using a Superose 6 10/300GL column; 0.5 ml fractions were collected, TCA-precipitated and loaded on SDS-PAGE for western blot analysis; immunoblots were probed with the antibodies indicated at the bottom or on the right side of the panels; the 37 KDa APE1 nuclear protein was used as control; **D**) Protein co-immunoprecipitation of Jurkat cell nuclear extracts. Immunoprecipitations were carried out with the antibodies indicated on top of each panel; these antibodies were specific for: IKAROS, the general P-TEFb activator BRD4, NuRD-associated proteins (Mi2, RBBP4, MTA2, MBD3) or P-TEFb components (CDK9, CYCT1). Immunoblots were probed with the antibodies indicated at the bottom of the panels. Input samples represent 2% of nuclear extracts; IgG: isotype-matched immunoglobulins; asterisk: IgG light chains; filled dots: non-specific bands; n≥3; **E**) The nuclear protein PCNA was used as negative control for the immunoprecipitation procedure; **F**) Summary of the most relevant protein-protein interactions; +: strong interaction; +/−: weak interaction; −: no interaction; nd: not determined.

Several studies have demonstrated physical interaction between IKAROS and NuRD [5], [17], [18], [36] or IKAROS and P-TEFb [23], [27]. To define how IKAROS coordinates chromatin remodeling and transcription elongation activities, we first conducted tandem immunoaffinity purification and mass spectrometry (LC-MS/MS) analysis in Jurkat cells expressing Flag- and HA-tagged IKAROS (Flag-HA-Ik) (Figures 1A, S1A). LC-MS/MS of Jurkat/Flag-HA-Ik eluates indicated that IKAROS associates with PP1α NuRD (Mi2, MTA2, RBBP4 and MBD3) and P-TEFb (CDK9, CYCT1) components (Table 1), whereas the P-TEFb inhibitor HEXIM1 and the negative elongation factor NELF were not amongst the purified proteins. In addition, HEXIM1 did not immunoprecipitate with IKAROS (Figure S1B), thus suggesting that IKAROS interacts with the elongation-competent and catalytically active P-TEFb.

**Table 1:** Immunoaffinity purification of Flag-HA-IKAROS complexes from Jurkat cells and their identification by LC-MS/MS analysis.

Unique Peptides	Total Peptides	Reference	Gene Symbol	AVG	Coverage	Complex
67	141	CHD4_HUMAN	CHD4 (Mi2β)	3.3291	31,64%	NuRD
66	127	CHD3_HUMAN	CHD3 (Mi2α)	3.1430	35,15%	NuRD
28	53	MTA2_HUMAN	MTA2	3.2056	48,95%	NuRD
26	47	MTA1_HUMAN	MTA1	2.8485	47,83%	NuRD
21	45	P66A_HUMAN	GATAD2A (p66α)	3.2509	48,34%	NuRD
19	36	HDAC1_HUMAN	HDAC1	3.2693	42,12%	NuRD
13	23	RBBP4_HUMAN	RBBP4	3.7420	47,53%	NuRD
11	23	MBD3_HUMAN	MBD3	3.2794	31,62%	NuRD
9	12	MBD2_HUMAN	MBD2	2.7871	24,82%	NuRD
8	14	PP1A_HUMAN	PPP1CA (PP1α)	2.7986	32,73%	P-TEFb
7	8	CDK9_HUMAN	CDK9	2.4313	17.47%	P-TEFb
3	3	CCNT1_HUMAN	CCNT1 (CYCLIN T1)	3.3421	5,65%	P-TEFb
25	97	IKZF1_HUMAN	IKZF1 (IKAROS)	3.4050	39,31%	
20	71	IKZF2_HUMAN	IKZF2 (HELIOS)	3.9466	33,65%	
10	11	IKZF3_HUMAN	IKZF3 (AIOLOS)	2.8839	25,74%	

The percentage values indicate the sequence coverage of the identified proteins; False Discovery Rate (FDR): 0%.

To determine whether IKAROS interactions with Mi2/NuRD and CDK9/P-TEFb define a single complex or multiple complexes, Flag-HA-Ik immunoaffinity-purified complexes were analyzed by size exclusion chromatography. Flag-HA-Ik co-fractionated with NuRD and P-TEFb components as a major peak of ∼2 MDa (Figure 1B). Additionally, Mi2, CDK9, CYCT1, PP1α and IKAROS co-eluted in nuclear extracts of wild type Jurkat cells (Figure 1C). Since IKAROS mainly eluted at 0.5 MDa (Figure 1C), it is likely that only a minority of the total nuclear IKAROS stably associates with the NuRD-P-TEFb complex. Accordingly, silver staining of IKAROS-purified complexes (Figure 1A) allowed the detection of two weak, but above-background, protein bands migrating at the range of the two known CDK9 isoforms [37] (Figure S1C). That only a small fraction of the nuclear CDK9/P-TEFb complex is highly active and capable of stimulating transcription elongation rate has also been shown in different systems [38], [39].

Next, we investigated whether the interaction of IKAROS with CDK9 or Mi2 could be differently modulated by fluctuation of IKAROS protein levels. Protein co-immunoprecipitation (co-IP) experiments were carried out in *Ikaros* knock-down Jurkat cells (Figure S1D). Co-IP results indicated that IKAROS-CDK9 interaction is more sensitive to IKAROS levels than the IKAROS-Mi2 interaction because IKAROS-CDK9 interaction is impaired in *Ikaros* knock-down Jurkat cells whereas IKAROS-Mi2 interaction is comparable in non-target as well as *Ikaros* knock-down Jurkat cells (Figure S1E). This suggests that in hematopoietic cells a fraction of nuclear IKAROS proteins can be dynamically assembled in a multifunctional protein complex containing both NuRD and P-TEFb components and that formation and/or stability of IKAROS-P-NuRD-P-TEFb interaction is influenced by the expression level of IKAROS.

Protein interactions between NuRD and P-TEFb components were then assessed by protein co-IP of Jurkat nuclear extracts. The NuRD-associated proteins Mi2, RBBP4, MTA2, and MBD3 interacted with the P-TEFb components CDK9 and CYCT1 (Figure 1D, S1F), whereas none of these factors interacted with the negative control nuclear protein PCNA (Figures 1D, E). Among the NuRD-associated proteins, only RBBP4 weakly interacted with the general P-TEFb activator BRD4 [40] (Figures 1D, S1G). CDK9, CYCT1, Mi2, MTA2, RBBP4, and MBD3 also co-immunoprecipitated with IKAROS (Figure 1D). DNase1 did not affect Mi2-CDK9 protein interaction (Figure S1H), indicating that DNA is dispensable. Thus, tandem immunoaffinity purification, size-exclusion chromatography and co-IP experiments demonstrate that IKAROS can assemble with the newly defined NuRD-P-TEFb multifunctional complex (Figure 1F).

### The simultaneous recruitment of P-TEFb and NuRD is IKAROS dose-dependent

**Figure 2:**
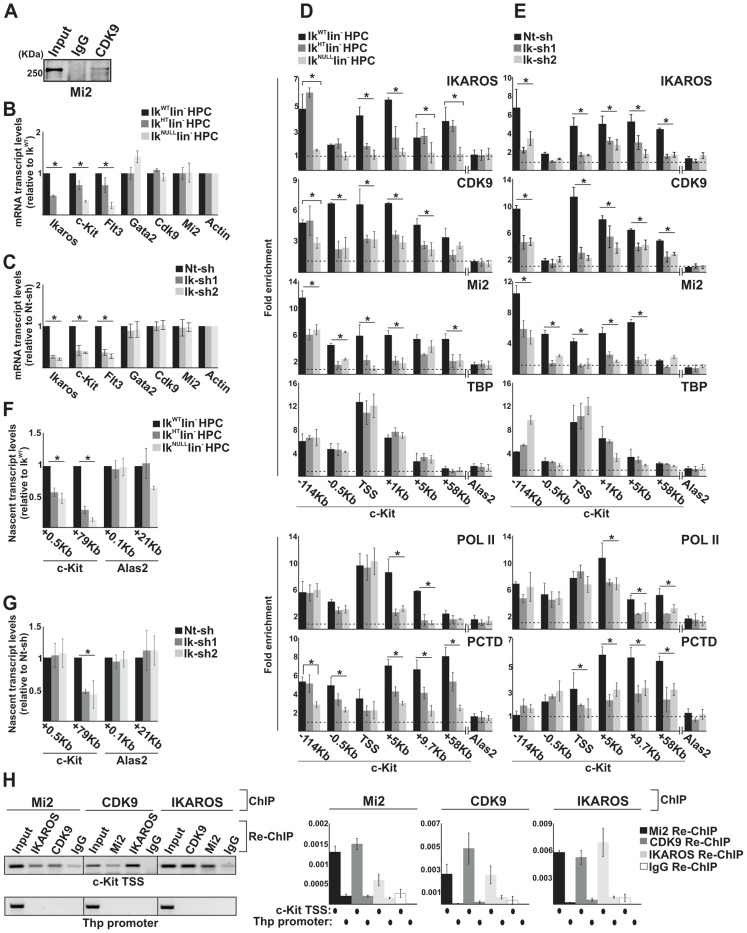
The simultaneous recruitment of P-TEFb and NuRD is IKAROS dose-dependent. **A**) Protein co-immunoprecipitation of G1E2 total cell lysates. Immunoprecipitations were carried out with CDK9 antibodies; immunoblots were probed with Mi2 antibodies; Input samples represent 2% of protein extracts; **B, C**) Gene expression profiles. mRNA samples were retro-transcribed with oligo-dT nucleotides; cDNA was used as template for qPCR with gene-specific primer sets; β-*Actin* was used as internal control; *y* axis: relative mRNA transcript enrichment levels; ratios are plotted as the mean ± Standard Deviation (SD) of the measurements; n≥4; **D, E**) Chromatin Immunoprecipitation (ChIP). ChIP assays were carried out with the antibodies indicated on the top of each panel; POL II: is an antibody against the N-terminal region of the large subunit of POL II and binds POL II in a phosphorylation-independent manner; PCTD: is an antibody against the CTD repeats phosphorylated at Ser2; *y*-axis: fold enrichments of *c-Kit* (−114 Kb enhancer; −0.5 Kb; TSS; +1 Kb, +5 Kb, +9.7 Kb, +58 Kb ORF regions) or *Alas2* (TSS; used as negative control) regions relative to *Thp* promoter and input samples are plotted as the mean ± SD of the measurements; a value of 1 (dotted lines) indicates no enrichment. Lin^−^ HPCs: bone marrow-derived lineage negative hematopoietic progenitor cells; Ik^WT^ lin^−^: *Ikaros* wild type lin^−^ HPCs; Ik^HT^ lin^−^: *Ikaros* heterozygote null lin^−^ HPCs; Ik^NULL^ lin^−^: *Ikaros* homozygote null lin^−^ HPCs; Nt-sh: non-target sh-RNA G1E2 clones; Ik-sh: *Ikaros*-specific sh-RNA (Ik-sh1 and Ik-sh2) G1E2 clones. *: *P*≤0.05 by Student's *t*-test; **F, G**) Gene expression profiles. RNA samples were retro-transcribed with random oligonucleotides to amplify nascent transcripts, which were used as templates for qPCR with intron-specific *c-Kit* (+0.5 Kb and +79 Kb regions), *Alas2* (+0.1 Kb and +21 Kb regions) or *Gapdh* (used as internal control) primer sets; *y* axis: relative nascent transcript enrichment levels; ratios are plotted as the mean ± Standard Deviation (SD) of the measurements; n≥4; **H**) Sequential ChIP (re-ChIP) assays carried out on G1E2 cells. Mi2, CDK9 or IKAROS antibodies were used for the first ChIP; IKAROS, CDK9, Mi2 antibodies or IgG controls were used for the second ChIP; left panel: representative examples of semi-quantitative PCR of re-ChIP sample carried out with primer sets specific for *c-Kit* TSS or *Thp* promoter; right panel: quantitative re-ChIP analysis carried out with the percent input method; re-ChIP samples were used as templates for quantitative PCR with primer sets specific for *c-Kit* TSS or *Thp* promoter; *y*-axis: fold enrichments of *c-Kit* TSS or *Thp* promoter (used as negative control) regions in re-ChIP samples relative to input samples are plotted as the mean ± SD of the measurements; n = 3.

We examined whether IKAROS could be a licensing factor for NuRD-P-TEFb complex recruitment to chromatin and whether the variation of IKAROS thresholds, which naturally occurs during hematopoiesis [5], [15], [41], could influence NuRD-P-TEFb association with IKAROS and thereby, regulate transcription elongation. To clarify this issue we studied two IKAROS-target genes, *c-Kit* and *Flt3* (Figure S2A), which are cell receptors critical for HPC survival that are downregulated in Ik^NULL^ HPCs [42]. We used: (i) bone marrow lineage negative (lin^−^) HPCs obtained from *Ikaros* wild type (Ik^WT^), *Ikaros* heterozygote null (Ik^HT^) or *Ikaros* homozygote null (Ik^NULL^) mice [6]; and (ii) the mouse G1E2 cells, which are proliferating hematopoietic progenitor-like cells [43]. The expression level of IKAROS in G1E2 cells was sufficient to detect Mi2-CDK9 interaction (Figure 2A).

*c-Kit* and *Flt3* expression correlated with the level of *Ikaros* expression in lin^−^ HPCs (Figure 2B). Similarly, the expression level of *c-Kit* and *Flt3* was significantly decreased in *Ikaros* knock-down G1E2 cells (Figure 2C). However, the expression level of additional *c-Kit* regulators, such as *Gata2* [44], *Cdk9* and *Mi2* (see below) was not reduced in Ik^NULL^ or *Ikaros* knock-down G1E2 cells (Figures 2B, C and S2B).

To investigate whether reduced *c-Kit* expression is a direct effect of the *Ikaros* loss, we carried out chromatin immunoprecipitation (ChIP) assays. ChIP experiments indicated that IKAROS was recruited to critical regulatory regions of the *c-Kit* locus [44] *e.g.*, the −114 Kb enhancer, the *c-Kit* Transcriptional Start Site -TSS-, and +1 Kb, +5 Kb as well as +58 Kb regions of the Open Reading Frame (ORF) in Ik^WT^ lin^−^ HPCs and G1E2 cells (Figure 2D, E). The same regions were occupied by CDK9, and Mi2 in Ik^WT^ lin^−^ HPCs, whereas the reduced level of IKAROS affected CDK9 and Mi2 recruitment at *c-Kit* (Figure 2D, E) but not at *c-Fos* promoter (Figure S2C), which was selected since *c-Fos* gene is expressed in HPCs but it is not regulated by IKAROS [45]. ChIP performed with TBP or POL II specific antibodies revealed that the PIC is assembled at *c-Kit* TSS regardless of *Ikaros* expression level (Figure 2D, E). However, the recruitment of POL II and POL II with phosphorylated Ser2 at the C-terminal domain (PCTD) at *c-Kit* ORF was reduced in Ik^HT^, Ik^NULL^ lin^−^ HPCs as well as the *Ikaros* knock-down G1E2 cells (Figure 2D, E), suggesting that IKAROS can positively control *c-Kit* transcription elongation. This assumption was supported by the significant decrease of CDK9 binding to *c-Kit* TSS and ORF (Figure 2D, E) and the consequent reduction of *c-Kit* nascent transcript levels towards the 3′ end of the gene (Figure 2F, G) in *Ikaros*-deficient cells.

The correlation between IKAROS expression level and the release of promoter-proximal paused POL II was further demonstrated by the analysis of *c-Kit* traveling ratio, which is determined by the relative ratio of POL II density in gene ORF *vs.* promoter-proximal regions [46], [47]. As indicated in Table 2, *c-Kit* traveling ratio decreased in Ik^HT^ and Ik^NULL^ lin^−^ HPCs, when compared to Ik^WT^.

**Table 2:** *c-Kit* POL II traveling ratio.

	Ik^WT^	Ik^HT^	Ik^NULL^	DMSO	Fvp
+5/−0.5	2.1	0.9	1	1.9	0.8
+9.7/−0.5	1.4	0.5	0.3	1.6	0.3

**Figure 3:**
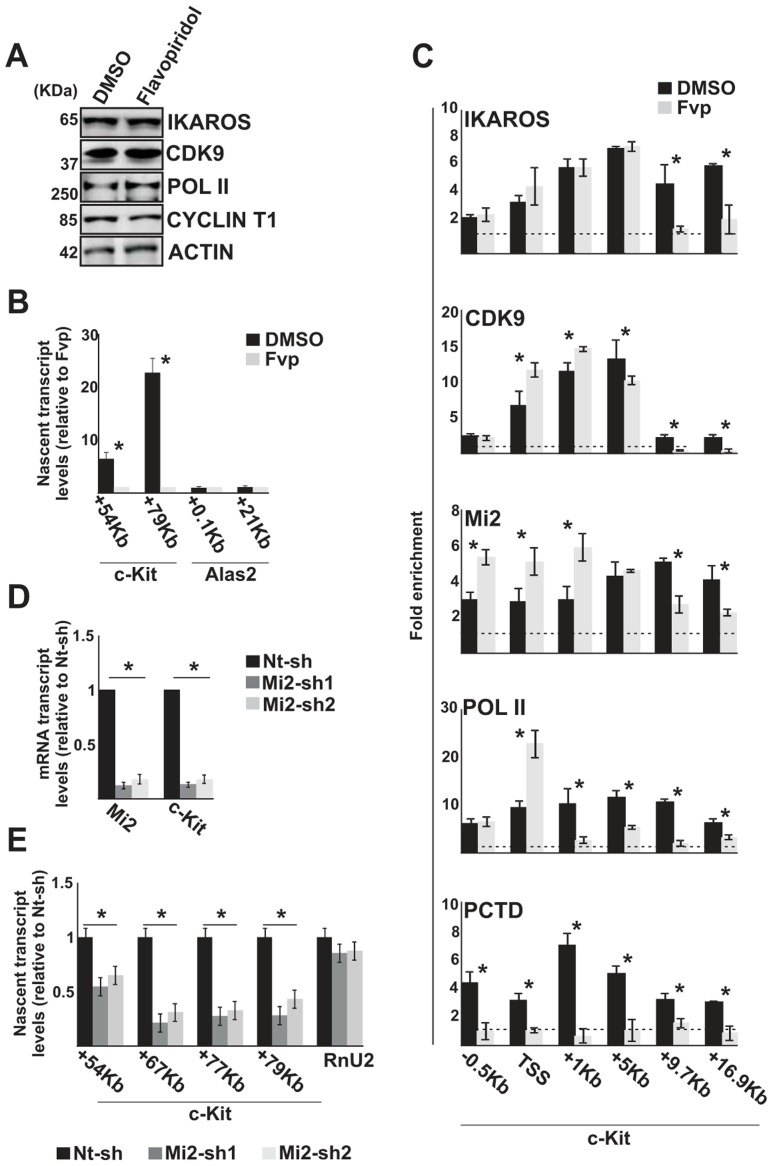
The IKAROS-NuRD-P-TEFb complex assists POL II during transcription elongation. In panels A–C, G1E2 cells were treated for 2 h either with 0.01% DMSO (DMSO) or 100 nM Flavopiridol (Fvp). **A**) Protein expression analysis. Western blot assays of total cell lysates of DMSO- or Flavopiridol-treated G1E2 cells; ACTIN was used as an internal control; the antibodies used are indicated on the right side of the panels; **B, E**) Gene expression profiles. RNA samples were retro-transcribed with random oligonucleotides to amplify nascent transcripts, which were used as templates for qPCR with intron-specific *c-Kit* (+54 Kb, +67 Kb, +77 Kb, +79 Kb regions) or *Alas2* (+0.1 Kb, +21 Kb regions) primer sets; *Rnu2-1* (a Flavopiridol-insensitive gene) was used as internal control in panel B; *Gapdh* was used as internal control in panel E; *y* axis: relative nascent transcript enrichment levels; ratios are plotted as the mean ± Standard Deviation (SD) of the measurements; n≥4; **C**) ChIP assays were carried out with the antibodies indicated on the top of each panel; POL II: is an antibody against the N-terminal region of the large subunit of POL II and binds POL II in a phosphorylation-independent manner; PCTD: is an antibody against the CTD repeats phosphorylated at Ser2; *y*-axis: fold enrichments of *c-Kit* regions (−.5 Kb; TSS, +0.5 Kb, +1 Kb, +5 Kb, +9.7 Kb, +16.9 Kb ORF regions) relative to *Thp* promoter and input samples are plotted as the mean ± SD of the measurements; a value of 1 (dotted lines) indicates no enrichment; n≥4; **D**) Gene expression profiles. mRNA samples were retro-transcribed with oligo-dT nucleotides; cDNA was used as template for qPCR with gene-specific primer sets; *β-Actin* was used as internal control; *y* axis: relative mRNA enrichment levels; ratios are plotted as the mean ± SD of the measurements; n≥4. *: *P*≤0.05 by Student's *t*-test.

*c-Kit* traveling ratio values (as defined by the relative ratio of POL II density in gene ORF *vs.* promoter-proximal regions) were obtained by chromatin immunoprecipitation with POL II antibody, which recognizes the N-terminal region of the large subunit of POL II and binds POL II in a phosphorylation-independent manner and indicate the enrichment levels of *c-Kit* +5/−0.5 or +9,7/−0.5 regions relative to the control and the input samples (see also Figure 2 and 3 legends); Ik^WT^: *Ikaros* wild type HPCs; Ik^HT^: *Ikaros* heterozygote null HPCs; Ik^NULL^: *Ikaros* homozygote null lin^−^ HPCs; DMSO: Dimethyl sulfoxide-treated G1E2 cells (0.01% for 2 h); Fvp: Flavopiridol-treated G1E2 cells (100 nM for 2 h).

To address whether IKAROS, CDK9 and Mi2 simultaneously co-occupy the *c-Kit* TSS, sequential ChIP (re-ChIP) [48] was carried out. Sequential ChIP analysis provided the evidence that in hematopoietic progenitors, these proteins can associate to the *c-Kit* TSS together (Figure 2H), hence suggesting that transcription elongation is promoted by the IKAROS-mediated recruitment of the NuRD-P-TEFb complex.

Collectively, these results indicate that in HPCs, IKAROS facilitates productive transcription elongation at *c-Kit* (Figure 2) and *Flt3* (Figure S2D, E; Table S1) genes and that reduced IKAROS levels, which do not affect the PIC assembly, can perturb transcription elongation.

### The IKAROS-NuRD-P-TEFb complex assists POL II during transcription elongation

Since IKAROS, CDK9 and Mi2 were detected at *c-Kit* TSS and ORF in Ik^WT^ HPCs (Figure 2D, E), we tested whether these factors play an active role during transcription elongation or associate with chromatin at *c-Kit* ORF independently of productive transcription elongation. Thus, transcription elongation was inhibited with Flavopiridol, which is a specific inhibitor of CDK9 activity [49]. G1E2 cells were treated with 100 nM Flavopiridol, for a short period of time (2 hours) to avoid indirect effects due to general inhibition of gene expression [50]. As expected, CDK9, POL II and CYCT1 protein levels were not reduced in G1E2-treated cells (Figure 3A), and IKAROS phosphorylation [35] appeared to be unmodified (Figure S3A). However, *c-Kit* and *Flt3* nascent transcript levels were significantly reduced (Figures 3B, S3B). Flavopiridol treatment of G1E2 cells did not affect IKAROS recruitment to the *c-Kit* (Figure 3C) or *Flt3* (Figure S3C) promoter region. Furthermore, decreased detection of POL II within the gene ORF, POL II accumulation at *c-Kit* TSS, and lack of PCTD association at *c-Kit* TSS and ORF, confirmed that Flavopiridol did not affect POL II loading, hence PIC assembly (Figures 3C, S3C) but substantially reduced transcription elongation (Table 2). Similar results (reduced *c-Kit* expression, unchanged IKAROS recruitment and lack of PCTD association to *c-Kit* promoter) were obtained in *Cdk9* knock-down G1E2 cells (Figure S3D–F). Thus, transcription elongation block (obtained either by Flavopiridol treatment or *Cdk9* knock-down) does not affect loading of IKAROS, CDK9/P-TEFb and Mi2/NuRD at *c-Kit* TSS region, but impedes P-TEFb and, surprisingly, IKAROS and NuRD recruitment within the gene ORF.

Since the elongation block by Flavopiridol led to an accumulation of Mi2 at *c-Kit* TSS and reduced association with the downstream regions (Figure 3C), we assessed whether NuRD could directly influence transcription elongation by knock-down of one of the NuRD components, Mi2, in G1E2 cells. The *Mi2* knock-down cells showed a reduction of *c-Kit* and *Flt3* mature as well as nascent transcripts (Figure 3D, E). The fact that *Mi2* knock-down had a greater effect on transcripts generated at distal rather than proximal regions, suggests that the NuRD complex can assist and facilitate POL II progression during transcription elongation.

### IKAROS contributes to PP1α association with the NuRD-P-TEFb complex

**Figure 4:**
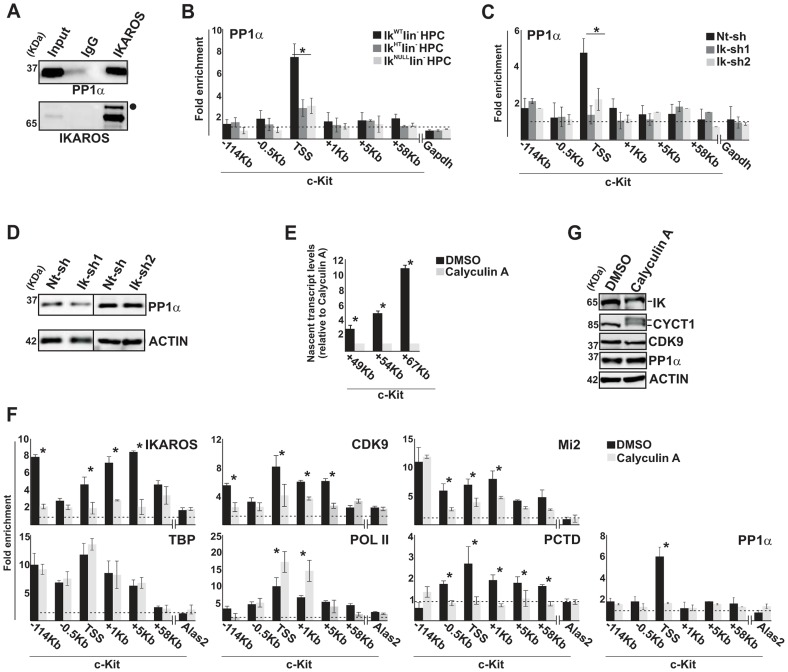
IKAROS contributes to PP1α association with the NuRD-P-TEFb complex. **A**) Protein co-immunoprecipitation of G1E2 nuclear extracts. Immunoprecipitations were carried out with IKAROS antibodies or isotype-matched immunoglobulins (IgG) and immunoblots were probed with PP1α or IKAROS antibodies; Input samples represent 2% of nuclear extracts; filled dot: non-specific band; **B, C, F**) ChIP assays were carried out with the antibodies labeled on the top of each panel; POL II: is an antibody against the N-terminal region of the large subunit of POL II and binds POL II in a phosphorylation-independent manner; PCTD: is an antibody against the CTD repeats phosphorylated at Ser2. Lin^−^ HPCs: bone marrow-derived lineage negative hematopoietic progenitor cells; Ik^WT^ lin^−^: *Ikaros* wild type lin^−^ HPCs; Ik^HT^ lin^−^: *Ikaros* heterozygote null lin^−^ HPCs; Ik^NULL^ lin^−^: *Ikaros* homozygote null lin^−^ HPCs; Nt-sh: non-target sh-RNA G1E2 clones; Ik-sh: *Ikaros*-specific sh-RNA (Ik-sh1 and Ik-sh2) G1E2 clones; DMSO: G1E2 cells treated for 30 min with 0.05% DMSO (diluent control); Calyculin A: G1E2 cells treated for 30 min with100 nM Calyculin A; *y*-axis: fold enrichments of *c-Kit* (−114 Kb; −0.5 Kb; TSS; +1 Kb, +5 Kb, +58 Kb ORF regions), *Alas2* (TSS) or *Gapdh* (TSS) regulatory regions relative to *Thp* promoter and input samples are plotted as the mean ± Standard Deviations (SD); a value of 1 (dotted lines) indicates no enrichment; n≥4; *Alas2*, *Gapdh* and *Thp* TSS regions were used as negative controls; **D, G**) Protein expression analysis. Western blot assays of total cell lysates were performed with non-target (Nt-sh) or *Ikaros*-specific (Ik-sh1 and Ik-sh2) sh-RNA G1E2 clones and with DMSO- or Calyculin A-treated G1E2 cells; the antibodies used are indicated on the right side of the panels; **E**) Gene expression profiles. RNA samples were retro-transcribed with random oligonucleotides to amplify nascent transcripts, which were used as templates for qPCR with intron-specific *c-Kit* (+49 Kb, +54 Kb, +67 Kb regions) or *Gapdh* (used as internal control) primer sets; *y* axis: relative nascent transcript enrichment levels; ratios are plotted as the mean ± SD of the measurements; n≥4. *: *P*≤0.05 by Student's *t*-test.

IKAROS is a substrate of PP1 activity [35] and PP1α can act as a positive regulator of CDK9/P-TEFb [29]. IKAROS interacts with the α catalytic subunit of PP1 in G1E2 cells (Figure 4A), in COS-7 cells transfected with IKAROS (Figure S4A) and in Jurkat cells (Table 1). Among the PP1 catalytic subunits, we identified PP1α as the most relevant and abundant interacting partner of IKAROS (Table S2). Thus, we investigated whether, by binding to PP1α and CDK9, IKAROS could deliver PP1α to the large P-TEFb complex, thereby activating CDK9/P-TEFb and favoring transcription elongation. In accordance with this assumption, PP1α recruitment was demonstrated at *c-Kit* TSS in Ik^WT^ and much less efficiently in Ik^HT^ or Ik^NULL^ lin^−^ HPCs (Figure 4B). PP1α recruitment to *c-Kit* TSS also decreased in *Ikaros* knock-down G1E2 clones (Figure 4C), even though PP1α expression was similar to control cells (Figure 4D).

The role of the IKAROS-PP1α interaction for CDK9/P-TEFb activation was further delineated by exploring the influence of Calyculin A, a PP1/PP2A inhibitor [34], on *c-Kit* and *Flt3* transcription elongation. Short-term treatment with Calyculin A did not cause toxic effects or cell death (Figure S4B), but induced significant reduction of *c-Kit* nascent transcripts (Figure 4E), whereas transcription elongation was not reduced upon treatment with inhibitors specific for PP2A (Okadaic Acid) or PP2B (Cyclosporin A) (Figure S4C, D), which are two phosphatases that do not dephosphorylate IKAROS proteins [35]. ChIP assay revealed that IKAROS was not significantly recruited to the *c-Kit* locus in Calyculin A-treated cells (Figure 4F). In general, CDK9, Mi2 and PCTD occupancy at *c-Kit* TSS, +1 and +5 regions, and PP1α recruitment at *c-Kit* TSS were also diminished, whereas POL II accumulated at TSS and +1 region and TBP recruitment did not vary (Figure 4F). Similar outcomes were observed at the *Flt3* locus (Figure S4E–H). Calyculin A treatment did not affect CDK9 or PP1α protein levels, but rather led to the appearance of two slow-migrating CYCT1- and IKAROS-specific bands (Figure 4G). These bands, which are likely corresponding to phosphorylated forms of CYCT1 and IKAROS proteins, were expressed at detectable levels. Thus, it can be concluded that the IKAROS-PP1α interaction: (i) contributes to IKAROS dephosphorylation and efficient binding to chromatin; (ii) allows PP1α association with *c-Kit* and *Flt3* TSS and (iii) promotes CDK9/P-TEFb activation in order to release promoter-proximal paused POL II.

### The IKAROS-PP1 interaction is required for normal hematopoietic cell differentiation

**Figure 5:**
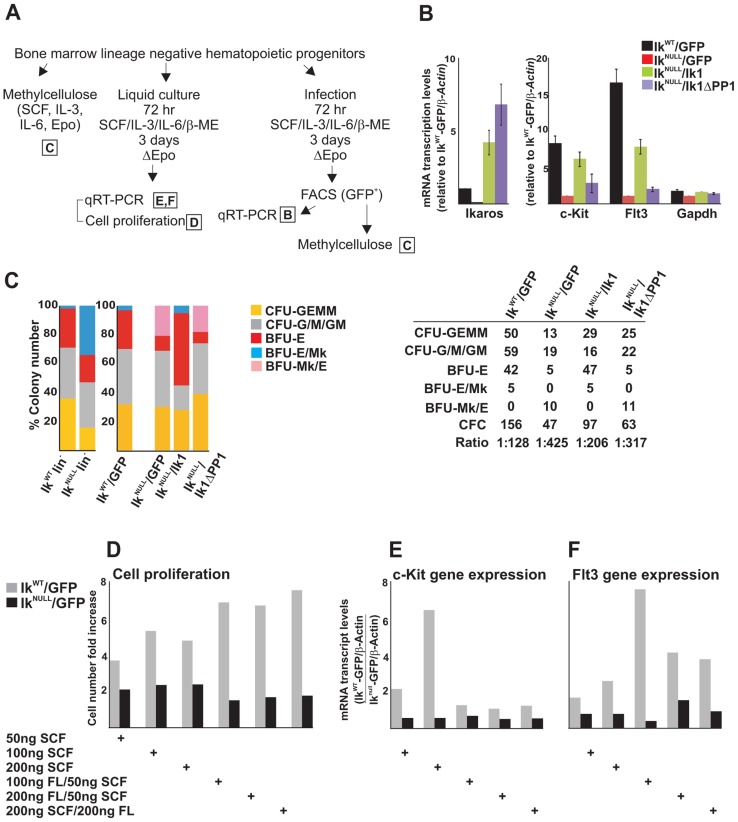
The IKAROS-PP1 interaction is required for normal hematopoietic differentiation. *Ikaros* homozygote null (Ik^NULL^) bone marrow-derived lineage negative (lin^−^) hematopoietic progenitor cells (HPCs) were transduced with pMSCV/Ik (Ik^NULL^/Ik), pMSCV/IkΔPP1 (Ik^NULL^/IkΔPP1) or the empty pMSCV vector (Ik^NULL^/GFP); *Ikaros* wild type (Ik^WT^) lin^−^ HPCs were transduced with the empty pMSCV vector only (Ik^WT^/GFP); **A**) Scheme of the experimental procedure; Epo: Erythropoietin; ΔEpo: absence of Erythropoietin; **B**) Gene expression profiles in lin^−^ transduced HPCs; mRNA samples were retro-transcribed with oligo-dT nucleotides; cDNA was used as templates for qPCR with *Ikaros*, *c-Kit*, *Flt3* or *Gapdh* specific primer sets; *β-Actin* was used as internal control; *y* axis: relative mRNA enrichment levels; ratios are plotted as the mean ± SD of the measurements; n = 3. *: *P*≤0.05 by Student's *t*-test; **C**) *In vitro* hematopoietic differentiation of lin^−^ HPCs. Clonogenic assays in methylcellulose of uninfected (Ik^WT^ lin^−^ and Ik^NULL^ lin^−^) or transduced (Ik^WT^/GFP; Ik^NULL^/GFP; Ik^NULL^/Ik; Ik^NULL^/IkΔPP1) lin^−^ HPCs; cells were seeded on methylcellulose and colonies were scored at day 14; CFU-GEMM: colony forming unit granulocyte, erythrocyte, macrophage, megakaryocyte; CFU-G/M/GM: collectively identifies granulo-macrophage colonies, BFU-E: burst-forming unit erythrocyte; BFU-E/Mk: immature erythroid colonies with elevated megakaryocytic content; BFU-Mk/E: almost pure megakaryocyte colonies that contain only few clusters of immature erythroid cells; the data shown are representative of three independent experiments; table: absolute number of methylcellulose colonies; CFC: Colony Forming Cell; Ratio: ratio of methylcellulose colonies per number of plated lin^−^ HPCs; **D–F**) Cell proliferation and gene expression profiles in Ik^WT^/GFP and Ik^NULL^/GFP transduced lin^−^ HPCs upon SCF (Stem Cell Factor) and FL (Flt3 ligand) treatment; Ik^WT^/GFP and Ik^NULL^/GFP transduced lin^−^ HPCs were treated for 3 days with SCF and/or FL at the concentrations indicated at the bottom of the panels; **D**: cell proliferation and cell viability was determined by Trypan blue staining; *y* axis: cell number fold increase at day 0 and day 3; **E, F**: gene expression profile studies were carried out as detailed in panel B using *β-Actin* as internal control; *y* axis: mRNA enrichment levels relative to Ik^NULL^-GFP/*β-Actin* values; the data shown in panels D–F are representative results of three independent experiments.

The functional role of IKAROS-PP1 interaction during hematopoiesis was further investigated by *ex vivo* assays in methylcellulose (Figure 5A). Ik^WT^ and Ik^NULL^ lin^−^ HPCs were transduced with the control pMSCV empty vector (Ik^WT^/GFP or Ik^NULL^/GFP); Ik^NULL^ lin^−^ HPCs were also transduced with pMSCV/Ik1 (*Ikaros1* isoform, identified as Ik^NULL^/Ik1 HPCs) or pMSCV/Ik1ΔPP1 (a mutant of *Ikaros1* carrying the A465/7 mutation, identified as Ik^NULL^/Ik1ΔPP1 HPCs). Ik1ΔPP1 is an IKAROS mutant unable to bind PP1 and therefore, resistant to dephosphorylation [35]. This mutant is reported to possess a shorter half-like than wild type IKAROS and thus, we assessed the stability of Ik1ΔPP1 in Ik^NULL^/Ik1ΔPP1 HPCs by single-cell immunofluorescence (IF) analysis. Since these transgenes possess a Flag-HA tag, IF analysis was carried out with anti-HA antibodies. IF studies indicated that Ik1ΔPP1 accumulates in the nucleus of Ik1ΔPP1-infected lin^−^ HPCs (Figure S5A). As well, in 293T transfected cells, whereby Ik1ΔPP1 was expressed at slightly lower levels than Ik1, Ik1ΔPP1 could interact with several known IKAROS protein partners (Mi2, CDK9, CYCT1 and MTA2) as demonstrated by protein co-IP experiments (Figure S5B).

In transduced lin^−^ HPCs, *Ikaros1* and *Ikaros1ΔPP1* transgene expression levels were about 5- and 8-fold higher than endogenous *Ikaros* (Figure 5B), which is comparable to other IKAROS rescue models [3]. As in Ik^NULL^ lin^−^ HPCs, *c-Kit* and *Flt3* expression was impaired in Ik^NULL^/GFP lin^−^ HPCs. Importantly, infection of Ik^NULL^ lin^−^ HPCs with pMSCV/Ik1 increased *c-Kit* expression to 74% and *Flt3* expression to 46.7% (if their expression levels in Ik^WT^/GFP lin^−^ HPCs is considered as 100%), whereas expression of *c-Kit* and *Flt3* genes was only increased to 33.3% (for *c-Kit*) and 11.5% (for *Flt3*) upon pMSCV/Ik1ΔPP1 infection (Figure 5B). Thus, even though Ik1ΔPP1 could slightly increase *c-Kit* and *Flt3* gene expression in lin^−^ transduced HPCs, Ik1 restored transcription elongation of these target genes more efficiently, indicating the functional importance of the IKAROS-PP1 interaction for gene regulation.

Clonogenic assays in methylcellulose revealed that Ik^NULL^ lin^−^ HPCs displayed decreased Colony Forming Cell (CFC) activity (1/166 Ik^NULL^ lin^−^ HPCs *vs.* 1/33 Ik^WT^ lin^−^ HPCs) as well as reduced colony size, which suggests reduction of cell proliferative potential. Ik^NULL^ lin^−^ HPCs produced less mixed CFC (CFU-GEMM) and increased erythroid/megakaryocyte (BFU-E/Mk) colonies (Figure 5C).

Although the expression of the c-KIT receptor at the cell surface of Ik^NULL^ HPCs is minimal compared to Ik^WT^ HPCs [42], we investigated whether a close-to-normal c-KIT and FLT3 activation could be obtained by the stimulation of Ik^NULL^ HPCs with increasing amounts of Stem Cell Factor (SCF or KIT ligand) as well as FLT3 ligand (FL). We observed that Ik^NULL^ lin^−^ HPCs were refractory to SCF and FL treatments. This functional limitation was demonstrated by the absence of: (i) induced cell proliferation (Figure 5D); (ii) *c-Kit* or *Flt3* induction (Figure 5E, F); and (iii) hematopoietic commitment and differentiation (Figure S5C) upon SCF and/or FL treatment of Ik^NULL^ lin^−^ HPCs. These results along with previously published work [51], suggest that IKAROS regulation of *c-Kit* and *Flt3* expression is critical for CFC activity and differentiation ability of HPCs.

To define the biological contribution of IKAROS-dependent control over transcription elongation, we studied the hematopoietic potential of Ik^NULL^ lin^−^ HPCs transduced with *Ikaros1* or the elongation-incompetent *Ikaros1ΔPP1* mutant. Ik^WT^/GFP lin^−^ HPCs maintained the same hematopoietic potential than non-transduced Ik^WT^ lin^−^ HPCs, both in term of colony number as well as colony types. However, Ik^NULL^/GFP lin^−^ HPCs had increased CFU-GEMM and BFU-Mk/E and reduced BFU-E potential compared to non-transduced Ik^NULL^ lin^−^ HPCs (Figure 5C; Ik^NULL^ *vs.* Ik^NULL^/GFP). The gain in CFU-GEMM might be caused by an advantageous transduction ability of multipotent progenitor cells, whereas the gain in megakaryocyte *vs.* erythroid component, as indicated by increased BFU-Mk/E colonies, might be related to the Erythropoietin deprivation during the infection procedure (Figure 5A). Infection of Ik^NULL^ lin^−^ HPCs with *Ikaros1* significantly augmented CFC activity of transduced HPCs (Figure 5C, table), which correlates with the increased number of CFU-GEMM, BFU-E/Mk and, moreover, BFU-E colonies (Figure 5C, table; Ik^NULL^ lin^−^ *vs.* Ik^NULL^/Ik1 lin^−^ HPCs). Interestingly, CFC activity of Ik^NULL^/Ik1ΔPP1 lin^-^ HPCs was lower than in Ik^NULL^/Ik1 lin^−^ HPCs (Figure 5C, table), but the hematopoietic potential of Ik^NULL^/GFP lin^−^ HPCs and Ik^NULL^/Ik1ΔPP1 lin^−^ HPCs was very similar (Figure 5C, table; Ik^NULL^/GFP lin^−^ *vs.* Ik^NULL^/Ik1ΔPP1 lin^−^ HPCs). Thus these results suggest that the *Ikaros1ΔPP1* mutant is defective in restoring hematopoietic functions and that IKAROS interaction with PP1, which is required for productive transcription elongation, is important for normal hematopoiesis.

## Discussion

P-TEFb can be recruited to gene promoters either by the general P-TEFb activator BRD4 [40] or by transcriptional activators that bind to specific consensus DNA sequences [37]. We previously reported that IKAROS acts as a template for the recruitment of P-TEFb to specific genes in hematopoietic cells [23], [27]. The partnership of IKAROS with the NuRD complex, which is critical for chromatin organization is also very-well established [5], [7], [18], [25]. The results presented here show that NuRD and P-TEFb can be associated together in a multifunctional complex, and that IKAROS is required for NURD-P-TEFb complex recruitment to specific genes in HPCs. Additionally, our results suggest that Mi2/NuRD actively participates in relieving POL II promoter-proximal pausing and contributes to the control of transcription elongation of IKAROS-target genes.

### IKAROS association with NuRD-P-TEFb complex: A combination of chromatin organization and transcription elongation activities

Size fractionation analysis of nuclear extracts and IKAROS immunoaffinity-purified protein complexes revealed that a modest but significant percentage of nuclear CDK9 and CYCT1 (P-TEFb) is stably associated with IKAROS and NuRD components. This association resembles to the small and highly active portion of P-TEFb that is included in the Super Elongation Complexes (SECs), known to stimulate transcription elongation rate [38], [39]. Interestingly, based on LC-MS/MS analysis, it appears that several subunits of the SECs interact with IKAROS in hematopoietic cells (Table S3). Furthermore, the absence of the transcription elongation inhibitor, NELF, and the P-TEFb inhibitor, HEXIM1, among the IKAROS-NuRD-P-TEFb interacting proteins supports the notion that the newly defined complex favors transcription elongation in hematopoietic cells.

Nucleosomes form barriers for the elongating POL II. The main remodeling complex known to facilitate POL II passage through nucleosomes is the histone chaperone complex FACT [52]. Although we cannot exclude that FACT is implicated together with IKAROS and the NuRD-P-TEFb complex in the control of *c-Kit* elongation, FACT components were not identified by LC-MS/MS analysis of IKAROS-associated proteins. Instead, our data indicate that POL II release and the rate of transcription elongation of these genes are affected by Mi2 knockdown. In addition, Mi2/NuRD occupancy at *c-Kit* and *Flt3* ORF decreased when transcription elongation was blocked by the CDK9 inhibitor Flavopiridol. Therefore, our results suggest that in HPCs, the NuRD complex can act as a chromatin complex that both destabilizes and restores nucleosomal structure in order to assist and facilitate the passage of POL II during transcription elongation. Accordingly, the NuRD complex can support nucleosome remodeling through the Mi2-associated helicase activity and histone deacetylation through the HDAC-associated activity [53], [54].

Initially regarded as a co-repressing complex because of its association with histone deacetylases and transcriptional repressors, the NuRD complex has also been associated with permissive chromatin and gene activation [5], [19]. More recently, it has been reported that the NuRD-mediated repression of a subset of pluripotency genes in ES cells occurs in a dynamic equilibrium with activation signals in order to fine-tune expression of these genes in response to differentiation stimuli [20]–[22]. Our findings bring a novel perspective to the role of NuRD in transcription regulation since we demonstrate a functional link between chromatin-associated activities that are required for the control of early transcription regulation *i.e.*, NuRD-dependent chromatin remodeling associated with gene priming and PIC assembly [55], and productive transcription elongation whereby Mi2/NuRD contributes to P-TEFb-dependent relief of paused POL II and transcription elongation.

### IKAROS recruits the NuRD-P-TEFb complex at chromatin in dosage-dependent manner

During differentiation of B-cell progenitors, the dynamic change of IKAROS expression level has been identified as a key mechanism for multiple target gene expression [15], [16]. Perturbation of IKAROS expression level has deleterious effects in double-positive thymocytes and in pre-B cells whereby reduced IKAROS function is associated with malignant transformation in mice and humans [41], [56], [57]. Furthermore, the IKAROS haploinsufficiency is reported to promote acute lymphoblastic leukemia with a high risk of relapse [58]. Our results suggest that chromatin association of the NuRD-P-TEFb complex to IKAROS target-genes depends on IKAROS expression level, hence providing a reasonable explanation for IKAROS dosage effects observed in hematological malignancies. In Ik^WT^ lin^−^ HPCs and G1E2 cells, a fraction of IKAROS associates with P-TEFb and NuRD and this complex is recruited to *c-Kit* and *Flt3* genes. In Ik^HT^ lin^−^ HPCs and *Ikaros* knockdown G1E2 cells, IKAROS, Mi2/NuRD and CDK9/P-TEFb are recruited less efficiently to gene regulatory regions and transcription elongation is affected although transcription initiation occurs normally and POL II is in a promoter-proximal paused configuration. Finally, in Ik^NULL^ HPCs, transcription initiation occurs normally but P-TEFb and NuRD recruitment to *c-Kit* and *Flt3* loci is impaired. Then, the enrichment of elongation-competent Ser2-phosphorylated POL II at gene ORF drops to almost background values. As a result, transcription elongation is profoundly reduced.

A large set of hematopoietic lineage-specific genes are characterized by permissive chromatin organization and PIC assembly at their promoters in HPCs [59]–[65]. IKAROS is required for the establishment of lineage-specific transcriptional programs [4], [5], [51]. Based on our results, we posit that when expressed at higher levels in HPCs and lymphoid progenitors, IKAROS induces rapid and dynamic expression of multiple genes poised for expression through the relief of the promoter-proximal paused POL II and transcription elongation. During alternative lineage-specification, IKAROS levels decrease, the IKAROS-NuRD-P-TEFb complex associates less efficiently to chromatin, and transcription elongation of different activated genes targeted by IKAROS declines.

### IKAROS-PP1α functional interaction

PP1α is involved in CDK9 dephosphorylation at Thr-186 and Ser-175 [30]. Dephosphorylation of Thr-186 and Ser-175 facilitates P-TEFb dissociation from the 7SK snRNP repressive complex. While the importance of Ser-175 phosphorylation/dephosphorylation is debated, dephosphorylation of Thr-186 is known to promote binding of the CDK9/P-TEF complex at genes TSS [29]. At the TSS, Thr-186 is phosphorylated by the TFIIH-associated CDK7, an event required for the catalytic activity of CDK9 (P-TEFb activation) and thus, the release of promoter-proximal paused POL II [66]. Hyperphosphorylation of IKAROS negatively affects its stability and DNA binding affinity [67], [68]. IKAROS dephosphorylation by PP1 [35] enhances IKAROS binding to DNA and, based on results presented here, it can also favor the transfer of this phosphatase to CDK9, thereby contributing to P-TEFb release from the 7SK snRNP and CDK9 activation [29]. Indeed, immunoaffinity purification and FPLC analyses suggest that PP1α associates with IKAROS, NuRD and P-TEFb in a 2 MDa complex that does not include subunits of the 7SK snRNP repressive complex. Since we found that PP1α is particularly abundant in close proximity to the TSS of the transcriptionally active *c-Kit* and *Flt3* genes, and PP1α recruitment to these TSS is highly influenced by IKAROS concentration, it can be argued that IKAROS contributes to chromatin recruitment of PP1α at the TSS of target genes hence, facilitating CDK9/P-TEFb association through dephosphorylation of CDK9 Thr-186 and possibly, Ser-175 [29], [69]. Furthermore, although CDK9 phosphorylation by CDK7 activates CDK9 and promotes POL II release, it has been demonstrated that imbalanced CDK9 hyperphosphorylation by CDK7 can have the opposite effect [30]. Thus, the relative higher abundance of PP1α at the TSS (when compared to the other regions bound by the IKAROS-NURD-P-TEFb) might be required to prevent excessive phosphorylation of TSS-bound CDK9 by the TFIIH-associated CDK7.

The importance of the IKAROS-PP1α network was demonstrated by results obtained with the PP1α inhibitor Calyculin A and with the Ik1ΔPP1 mutant. First, Calyculin A treatment, which leads to IKAROS hyperphosphorylation and reduced DNA binding [35], resulted in decreased recruitment of PP1α, Mi2 and CDK9 to *c-Kit* and *Flt3* promoters but did not affect TBP and POL II recruitment. Thus, PP1α activity is critical for IKAROS as well as NuRD and P-TEFb recruitment to these promoters and for transcription elongation, while being dispensable for PIC formation. Second, the rescue of Ik^NULL^ HPCs attempted with the Ik1ΔPP1 mutant, which interacts with Mi2, MTA2, CYCT1 and CDK9 (Figure S5B) but does not efficiently interact with PP1 [35], demonstrated that without the interaction with PP1, IKAROS cannot exert its normal function during hematopoiesis.

It is worth noting that PP1 can contribute to co-transcriptional pre-mRNA splicing control [70], [71], an event that could also favor transcription elongation of IKAROS target genes. In fact, LC-MS/MS analysis suggested that some proteins implicated in the process of transcription termination can interact with IKAROS in hematopoietic cells (Table S3). Based on our results we suggest that IKAROS, NuRD and P-TEFb are not only active at the TSS but they facilitate transcription elongation and could also influence transcription termination.

Thus, IKAROS control over transcription elongation at genes such as *c-Kit* and *Flt3* is likely to provide a rapid adjustment of their expression levels, and be a critical mechanism affecting HSC/HPCs interaction with the niche, cell fate and stress response [72]–[75].

**Figure 6:**
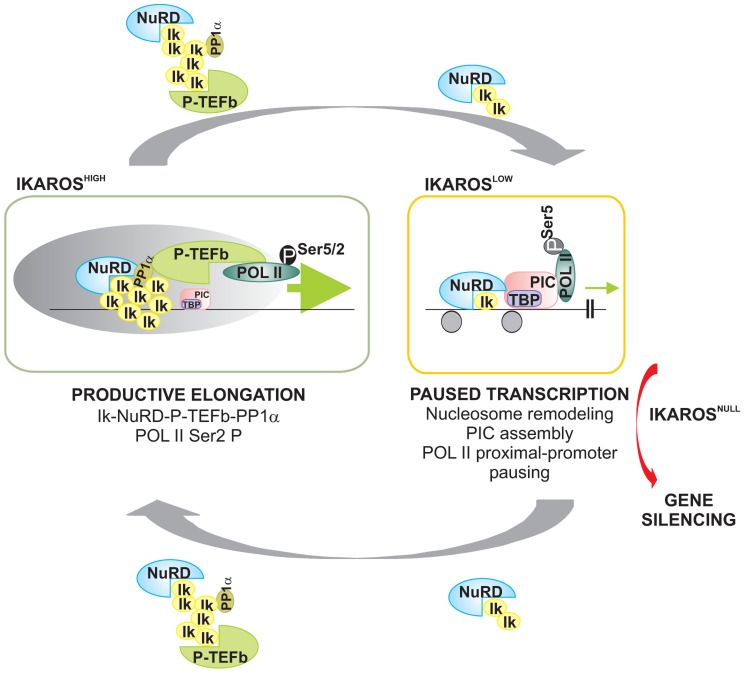
Model of IKAROS dosage effect for formation and recruitment of the NuRD-P-TEFb complex and transcription elongation control of IKAROS-target genes. *Productive elongation*: when expressed at high levels, as in lineage negative hematopoietic progenitor cells, IKAROS (Ik) associates Mi2/NuRD and CDK9/P-TEFb activities into one complex at gene transcription start site (TSS) and throughout the ORF of IKAROS-target genes. IKAROS dependent enrichment of PP1α at TSS provides a control to P-TEFb activation by the dephosphorylation of CDK9. Once activated, CDK9/P-TEFb phosphorylates Ser-2 on POL II CTD as well as NELF and DSIF, two negative regulators of POL II elongation and thereby, releases the promoter-proximal paused POL II. Then, IKAROS and the NuRD-P-TEFb complex assist POL II during elongation. Based on results obtained by LC-MS/MS analysis, the latter might occurs along with SEC subunits. *Paused transcription*: the gradual decrease of IKAROS protein concentration, such as normally observed at defined stages of hematopoietic cell differentiation, still allows Mi2/NuRD loading at gene regulatory regions as well as PIC formation at gene promoters. However, lack of P-TEFb and PP1α recruitment results in promoter-proximal pausing of POL II. *Gene silencing*: in absence of IKAROS, as observed in lymphoma and leukemia clones carrying mutated and/or deleted alleles of IKAROS, the IKAROS-target genes are embedded in inactive chromatin conformation (not depicted) and they are characterized by the absence of PIC formation. To simplify the model, some of the proteins or complexes mentioned are not indicated.

In conclusion, we have demonstrated that IKAROS is the DNA-binding subunit of the newly characterized NuRD-P-TEFb multifunctional complex, a chromatin-associated complex that contains chromatin remodeling (NuRD) as well as gene transcription elongation (P-TEFb) activities. IKAROS is important for CDK9 and Mi2 interaction and combined recruitment to actively transcribed genes in hematopoietic cells. We demonstrate that low IKAROS expression level does not preclude appropriate promoter organization but impairs productive elongation, whereas higher IKAROS levels are necessary to relieve promoter-proximal paused POL II and efficient transcription elongation of target genes (Figure 6). Our results suggest that NuRD can assist POL II transcription elongation complex throughout the ORF of IKAROS target genes and provides mechanistic cues to explain the central role played by IKAROS as transcriptional activator during hematopoietic lineage decision, differentiation, and interaction with the niche.

## Materials and Methods

### Protein co-IP, ChIP, re-ChIP, qPCR and qRT-PCR assays

These assays were done essentially as previously reported [7], [23], [27], [48].

### Fast Protein Liquid Chromatography (FPLC)

Protein complexes were purified from Jurkat nuclear extract or IKAROS immunoaffinity-purified complexes on an AKTA Purifier FPLC System (GE Healthcare) using a Superose 6 10/300GL column (GE Healthcare).

### Sequential immunoaffinity purification of IKAROS-associated proteins

Flag-HA sequential immunoaffinity (or Tap-Tag) purification was carried out as described in Nakatani *et al.* [76] starting from nuclear extracts prepared from pOZ-N-Flag-HA-IRES-ILR2 (mock sample) or pOZ-N-Flag-HA-IKAROS-IRES-ILR2- (Flag-HA-Ik sample) Jurkat cell clones. LC-MS/MS analysis was performed at the Taplin Mass Spectrometry Facility (Harvard University, Boston).

### Mouse transgenic lines and primary cell isolation

Homozygote (Ik^NULL^) and heterozygote (Ik^HT^) *Ikaros* null mice were genotyped by PCR as described [6]. Animals were sacrificed by cervical dislocation. Animal experiments were conducted in accordance with the Canadian Council on Animal Care (CCAC) guidelines and approved by the Maisonneuve-Rosemont Hospital animal care committee (approval numbers 2011-16 and 2011-17). Lineage negative (lin^−^) HPCs were purified using the easySep mouse hematopoietic progenitor enrichment kit (StemCell Technology).

### Retrovirus infection and clonogenic assays in methylcellulose

pMSCV/Ik1 and pMSCV/Ik1ΔPP1 vectors were generated by cloning the murine *Ikaros1* cDNA or the mutant *Ikaros1ΔPP1* cDNA, both with a Flag and HA tag at their N-terminal regions, into the MSCV-pgk-EGFP vector (Dr G. Sauvageau, IRIC). Retroviral infection of lin^−^ HPCs was carried out as described [77]. Lin^−^ HPCs were cultured for 3 days in medium containing 50 ng/ml SCF, 10 ng/ml IL3, 10 ng/ml IL6 and 5×10^−5^ M β-mercaptoethanol, without Erythropoietin. Transduced lin^−^/GFP^+^ HPCs were isolated on a FACS Aria III sorter (BD Biosciences) based on green fluorescence. HPCs were seeded in triplicate on complete methylcellulose medium (MethoCult Stemcell Technologies). Colonies were scored at day 14.

### Statistical analysis

Unpaired Student's *t*-test was used to determine the level of statistical significance (*P*-value).

Additional Materials and Methods information can be found in Text S1.

## Supporting Information

Figure S1 IKAROS expression and protein co-immunoprecipitation in Jurkat clones. Protein expression analysis. **A**) Western blot assays of total cell lysates of stably transfected Jurkat clones; Ik1 and Ik2 indicate the endogenous full-length and functional IKAROS isoforms [78]–[80]; Flag-HA-Ik indicates the double Flag and HA-tagged Ik2 protein; immunoblots were probed with IKAROS-specific antibody (left panel) or with an antibody directed against the HA tag (right panel); the Flag-HA-Ik protein is expressed at lower levels than the endogenous full-length isoforms; Jurkat mock: Jurkat cells expressing the pOZ-N-Flag-HA-IRES-ILR2 empty vector; Jurkat Flag-HA-IKAROS: Jurkat cells expressing the pOZ-N-Flag-HA-IRES-ILR2-IKAROS vector; **B**) Protein co-immunoprecipitation on increasing amount of G1E-2 whole cell extracts (WCE). Immunoprecipitations were performed with HEXIM1 antibodies or isotype-matched IgG control (IgG) as indicated at the top of the panels; immunoblots were probed with IKAROS or HEXIM1 antibodies; Input samples represent 2% of protein extracts; filled dot: non-specific bands; **C**) Purification of Flag-HA-Ik associated proteins. Nuclear extracts from Jurkat cells carrying the pOZ-N-Flag-HA-IKAROS-IRES-ILR2 vector and expressing a double tagged IKAROS (Flag-HA-Ik) or Jurkat cells expressing the pOZ-N-Flag-HA-IRES-ILR2 empty vector (Mock) were used for sequential immunoaffinity purification using Flag- followed by HA-conjugated matrix. A fraction of the purified complexes were loaded on SDS-PAGE and silver stained; E1: first HA elution; E2: second HA elution; MW: molecular weights (in KDa); putative CDK9_55_ and CDK9_42_ isoforms are indicated by arrows; compared to the Figure 1A, linear adjustment of contrast and brightness was applied by Photoshop software to the whole image; **D**) Protein expression analysis. Western blot assays of total cell lysates were performed with non-target (Nt-sh) or *Ikaros*-specific (Ik-sh) sh-RNA Jurkat clones; fold decrease of IKAROS protein levels in Ik-sh *vs.* Nt-sh samples was calculated as indicated at the bottom of the image, using ACTIN and PARP-1 as internal controls; densitometry analysis of Western blot gels was performed using Multi Gauge software (Fuji Film); **E**) Protein co-immunoprecipitation of whole cell extracts isolated from *Ikaros* knock-down (Ik-sh) or non-target control (Nt-sh) Jurkat cells. Immunoprecipitations were performed with the antibodies or isotype-matched IgG control (IgG) indicated at the top of the panels; immunoblots were probed with the antibodies indicated at the right-side of the panels; Input samples represent 2% of protein extracts; **F, G**) Protein co-immunoprecipitation of Jurkat cell nuclear extracts. Immunoprecipitations were performed with the antibodies or isotype-matched IgG control (IgG) indicated at the top of the panels; immunoblots were probed with the antibodies indicated at the right-side of the panels; Input samples represent 2% of protein extracts; asterisks: IgG heavy and light chains; **H**) Protein co-immunoprecipitation of Jurkat cell nuclear extracts performed with lysis buffer containing 1 µg/ml DNase1.
(TIF)

Figure S2 Factor recruitment to the *Flt3* locus in lineage negative hematopoietic progenitors and G1E2 cells. **A**) Schematic representation of the murine *c-Kit* and *Flt3* loci. Amplicon positions are indicated with arrows [44], [81]. For the *c-Kit* locus, they correspond to: −114 Kb enhancer region; −0.5 Kb region; the Transcriptional Start Site (TSS); +1 Kb, +5 Kb, +9.7 Kb, +16.9 Kb and +58 Kb Open Reading Frame (ORF) regions; for the *Flt3* locus, the TSS and the ORF +0.5 Kb, +2 Kb and +8.5 Kb regions; filled circles: TGGGAA IKAROS consensus binding sites that IKAROS specifically and directly binds in crude nuclear extracts of hematopietic cells [7], [79]; **B**) Protein expression analysis. Western blot assays of total cell lysates of *Ikaros* knock-down G1E2 clones; ACTIN was used as internal control; Nt-sh: non-target sh-RNA G1E2 clones; Ik-sh: *Ikaros*-specific sh-RNA (Ik-sh1 and Ik-sh2) G1E2 clones; **C–E**) Chromatin immunoprecipitation (ChIP). ChIP assays were carried out with the antibodies indicated on the top of each panel; POL II: is an antibody against the N-terminal region of the large subunit of POL II and binds POL II in a phosphorylation-independent manner; PCTD: is an antibody against the CTD repeats phosphorylated at Ser2; *y*-axis: fold enrichments of *Flt3* or *Alas2* regions relative to *Thp* promoter and input samples are plotted as the mean ± Standard Deviation (SD) of the measurements; a value of 1 (dotted lines) indicates no enrichment; n≥4; the amplicons tested recognized the *c-Fos* TSS, the *Flt3* TSS and ORF +2 Kb regions as well as the *Alas2* −1 Kb, TSS and +20 Kb ORF regions. Since the *Alas2* gene is not expressed in G1E2 cells, it was used as negative control; the Alas2 values shown in panel C correspond to the Alas2 values displayed in Figure 2D (CDK9 ChIP); lin^−^: bone marrow-derived lineage negative hematopoietic progenitor cells (HPCs); Ik^WT^ lin^−^: *Ikaros* wild type lin^−^ HPCs; Ik^HT^: *Ikaros* heterozygote null lin^−^ HPCs; Ik^NULL^ lin^−^: *Ikaros* homozygote null lin^−^ HPCs; Nt-sh: non-target sh-RNA G1E2 clones; Ik-sh: *Ikaros*-specific sh-RNA (Ik-sh1 and Ik-sh2) G1E2 clones. *: *P*≤0.05 by Student's *t*-test.
(TIF)

Figure S3 Factor recruitment to the *Flt3* and *c-Kit* loci in Flavopiridol-treated and *Cdk9* knock-down G1E2 cells. In panels A–C, G1E2 cells were treated for 2 h either with 0.01% DMSO (control) or 100 nM Flavopiridol. **A**) Protein co-immunoprecipitation of G1E2 total cell lysates. Immunoprecipitations were performed with an IKAROS-specific antibody and immunoblots were probed with the same antibody in order to exclude any significant variation of IKAROS phosphorylation levels upon Flavopiridol treatment; Input samples represent 2% of total cell lysates; filled dot: non-specific band; **B**) Gene expression profiles. RNA samples were retro-transcribed with random oligonucleotides to amplify nascent transcripts, which were used as template for qPCR with intron-specific *Flt3* (+0.5 Kb, +7 Kb regions) or *Alas2* (+2 Kb, +21 Kb regions, used as negative control) primer sets; *Rnu2-1*, a Flavopiridol-insensitive gene was used as internal control; *y* axis: relative nascent transcript enrichment levels; ratios are plotted as the mean ± Standard Deviation (SD) of the measurements; n≥4; **C, F**) Chromatin immunoprecipitation (ChIP). ChIP assays were carried out with the antibodies labeled on the top of each panel; POL II: is an antibody against the N-terminal region of the large subunit of POL II and binds POL II in a phosphorylation-independent manner; PCTD: is an antibody against the CTD repeats phosphorylated at Ser2; *y*-axis: fold enrichments of *Flt3* TSS or +2 and +8.5 ORF regions, *c-Kit* and *Alas2* TSS, relative to *Thp* promoter and input samples are plotted as the mean ± SD of the measurements; a value of 1 (dotted lines) indicates no enrichment; n≥4; **D**) Protein expression analysis. Western blot assays of total cell lysates of non-target (Nt-sh) or *Cdk9*-specific (*Cdk9*-sh1 and *Cdk9*-sh2) sh-RNA G1E2 clones; the antibodies used are indicated at the bottom of the panels; ACTIN was used as internal control; **E**) Gene expression profiles. mRNA samples were retro-transcribed with oligo-dT nucleotides; cDNA was used as template for qPCR with gene-specific primer sets; *β-Actin* was used as internal control; *y* axis: relative mRNA enrichment levels; ratios are plotted as the mean ± SD of the measurements; n≥4. *: *P*≤0.05 by Student's *t*-test.
(TIF)

Figure S4 IKAROS-PP1α interaction and factor recruitment to the *Flt3* locus in Calyculin A-treated G1E2 cells. **A**) Protein co-immunoprecipitation of COS-7 total cell lysates. COS-7 cells, which do not express any IKAROS protein, were transiently transfected with an expression vector encoding the murine *Ikaros1* cDNA with a Flag and HA tag at its N-terminal region (FH-Ik1); immunoprecipitations were performed with PP1α antibodies or isotype-matched IgG control (IgG); immunoblots were probed with antibodies directed against HA (for Flag-HA-Ik detection; upper panel) or PP1α (lower panel); Input samples represent 2% of nuclear extracts; **B**) CMX Ros staining of DMSO- (upper panels) or Calyculin A- (CalA, lower panels) treated G1E2 cells. Flow cytometry; FCS: Forward scatter; SSC: Side scatter; CMX Ros: red fluorescence emission from CMX Ros; **C, D, G**) Gene expression profiles of G1E2 cells. RNA samples were retro-transcribed with random oligonucleotides to amplify nascent transcripts, which were used as templates for qPCR with intron-specific *c-Kit* (+54 Kb, +67 Kb and +79 Kb regions), *Flt3* (+2 Kb and +67 Kb regions) or *Gapdh* (used as internal control) primer sets; *y* axis: relative nascent transcript enrichment levels; ratios are plotted as the mean ± Standard Deviations (SD) of the measurements; n≥4; in panel C, the experiments were performed on G1E2 cells treated for 30 min with 1 µM Okadaic Acid or its diluent as control; in panel D, the experiments were performed on G1E2 cells treated for 2 h with 1 µM Cyclosporin A or its diluent as control; in panel G, the experiments were performed on G1E2 cells treated for 30 min with 100 nM Calyculin A or its diluent as control; **E, F, H**) Chromatin Immunoprecipitation (ChIP). ChIP assays were carried out with the antibodies labeled on the top of each panel; POL II: is an antibody against the N-terminal region of the large subunit of POL II and binds POL II in a phosphorylation-independent manner; PCTD: is an antibody against the CTD repeats phosphorylated at Ser2; *y*-axis: fold enrichments of *Flt3* TSS or +2 Kb and +8.5 Kb ORF regions, *Alas2* TSS or *Gapdh* TSS regions (used as negative controls) relative to *Thp* promoter and input samples are plotted as the mean ± SD of the measurements; a value of 1 (dotted lines) indicates no enrichment; n≥4; lin^−^: bone marrow-derived lineage negative hematopoietic progenitor cells (HPCs); Ik^WT^ lin^−^: *Ikaros* wild type lin^−^ HPCs; Ik^HT^ lin^−^: *Ikaros* heterozygote null lin^−^ HPCs; Ik^NULL^ lin^−^: *Ikaros* null lin^−^ HPCs; Nt-sh: non-target sh-RNA G1E2 clones; Ik-sh: *Ikaros*-specific sh-RNA (Ik-sh1 and Ik-sh2) G1E2 clones. *: *P*≤0.05 by Student's *t*-test.
(TIF)

Figure S5 IKAROS1-ΔPP1 detection in hematopoietic progenitors and IKAROS1-ΔPP1 protein interactions. **A**) Single-cell immunofluorescence analysis. *Ikaros* homozygote null bone marrow-derived lineage negative hematopoietic progenitor cells (Ik^NULL^ lin^−^ HPCs) were transduced with pMSCV/Ik1 (Ik^NULL^/Ik1), pMSCV/Ik1ΔPP1 (Ik^NULL^/Ik1ΔPP1) or the empty pMSCV vector (Ik^NULL^/GFP) and then cytospun and fixed on slides; infected cells were recognized as GFP^+^ cells; IKAROS1 or IKAROS1ΔPP1 proteins were detected with mouse anti-HA and Texas Red (TR)-conjugated anti-mouse antibody; representative lin^−^ HPCs transduced cells are shown where infected cells are detected as green signals and IKAROS1- or IKAROS1ΔPP1-expressing cells are detected as red signals; **B**) Protein co-immunoprecipitation of 293T total cell lysates. 293T cells, which do not express any IKAROS protein, were transiently transfected with an expression vector encoding the murine *Ikaros1* cDNA (FH-Ik1), the mutant *Ikaros1ΔPP1* (FH-Ik1ΔPP1) cDNA or the empty vector (Mock), all with a Flag and HA tag at their N-terminal regions; immunoprecipitations were performed with FLAG antibodies; immunoblots were probed with IKAROS, Mi2, CDK9, CYCLIN T1 or MTA2 antibody; Input samples represent 2% of total cell lysates; **C**) *In vitro* hematopoietic differentiation of lineage negative (lin^−^) hematopoietic progenitor cells (HPCs). Clonogenic assays in methylcellulose of Ik^WT^ or Ik^NULL^ lin^−^ HPCs grown for 3 days in cytokine-supplemented liquid cultures; lin^−^ HPCs were seeded on methylcellulose and colonies were scored at day 14; CFU-GEMM: colony forming unit granulocyte, erythrocyte, macrophage, megakaryocyte; CFU-G/M/GM: collectively identifies granulo-macrophage colonies; BFU-E: burst-forming unit erythrocyte; BFU-E/Mk: immature erythroid colonies with elevated megakaryocytic content; BFU-Mk/E: almost pure megakaryocyte colonies that contain only few clusters of immature erythroid cells; CFU-DC: colony forming unit dendritic cell; the data shown are representative of three independent experiments.
(TIF)

Table S1 *Flt3* POL II traveling ratio. *Flt3* traveling ratio values (as defined by the relative ratio of POL II density in gene ORF *vs.* promoter-proximal regions) were obtained by chromatin immunoprecipitation with POL II antibody, which recognizes the N-terminal region of the large subunit of POL II and binds POL II in a phosphorylation-independent manner and indicate the enrichment levels of *Flt3* +2/TSS regions relative to the control and the input samples (see also Figure S2 and S3 legends); Ik^WT^: *Ikaros* wild type HPCs; Ik^HT^: *Ikaros* heterozygote null HPCs; Ik^NULL^: *Ikaros* homozygote null lin^−^ HPCs; DMSO: Dimethyl sulfoxide-treated G1E2 cells (0.01% for 2 h); Fvp: Flavopiridol-treated G1E2 cells (100 nM for 2 h).
(DOCX)

Table S2 Protein phosphatase 1 catalytic subunits identified by immunoaffinity purification and LC-MS/MS analysis of Flag-HA-IKAROS complexes. The percentage values indicate the sequence coverage of the identified proteins; False Discovery Rate (FDR): 0%.
(DOCX)

Table S3 Super Elongation Complex (SEC) and microprocessor components identified by immunoaffinity purification and LC-MS/MS analysis of Flag-HA-IKAROS complexes. False Discovery Rate (FDR): 0%.
(DOCX)

Text S1 Supplemental methods.
(DOCX)
