# Supplementary figures and images for: The IKAROS Interaction with a Complex Including Chromatin Remodeling and Transcription Elongation Activities Is Required for Hematopoiesis
Source: PLoS Genet. 2014 Dec 4;10(12):e1004827. doi: 10.1371/journal.pgen.1004827 (PMC4256266; doi:10.1371/journal.pgen.1004827)

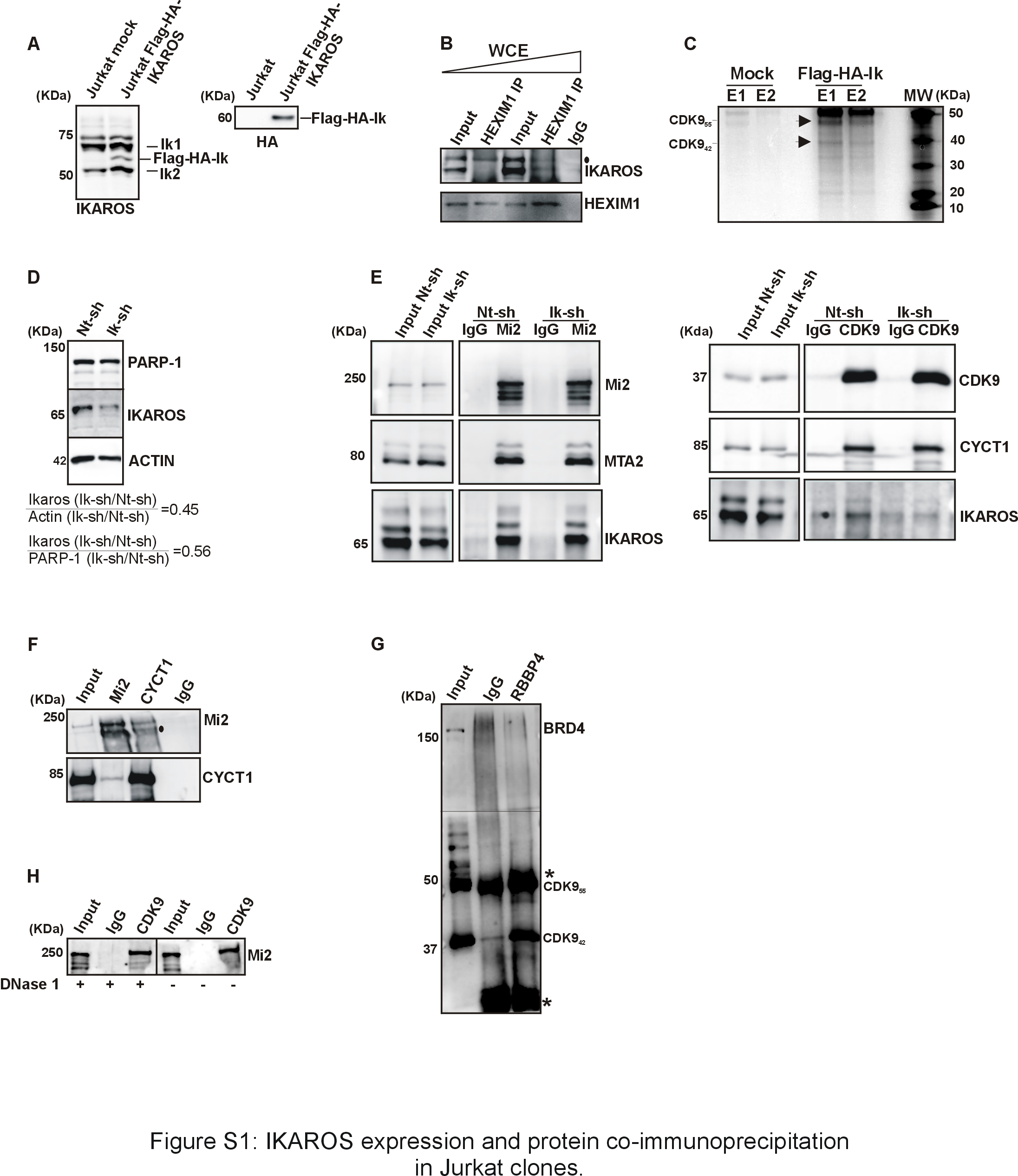

Supplement: Figure S1 — IKAROS expression and protein co-immunoprecipitation in Jurkat clones. Protein expression analysis. A) Western blot assays of total cell lysates of stably transfected Jurkat clones; Ik1 and Ik2 indicate the endogenous full-length and functional IKAROS isoforms [78]–[80]; Flag-HA-Ik indicates the double Flag and HA-tagged Ik2 protein; immunoblots were probed with IKAROS-specific antibody (left panel) or with an antibody directed against the HA tag (right panel); the Flag-HA-Ik protein is expressed at lower levels than the endogenous full-length isoforms; Jurkat mock: Jurkat cells expressing the pOZ-N-Flag-HA-IRES-ILR2 empty vector; Jurkat Flag-HA-IKAROS: Jurkat cells expressing the pOZ-N-Flag-HA-IRES-ILR2-IKAROS vector; B) Protein co-immunoprecipitation on increasing amount of G1E-2 whole cell extracts (WCE). Immunoprecipitations were performed with HEXIM1 antibodies or isotype-matched IgG control (IgG) as indicated at the top of the panels; immunoblots were probed with IKAROS or HEXIM1 antibodies; Input samples represent 2% of protein extracts; filled dot: non-specific bands; C) Purification of Flag-HA-Ik associated proteins. Nuclear extracts from Jurkat cells carrying the pOZ-N-Flag-HA-IKAROS-IRES-ILR2 vector and expressing a double tagged IKAROS (Flag-HA-Ik) or Jurkat cells expressing the pOZ-N-Flag-HA-IRES-ILR2 empty vector (Mock) were used for sequential immunoaffinity purification using Flag- followed by HA-conjugated matrix. A fraction of the purified complexes were loaded on SDS-PAGE and silver stained; E1: first HA elution; E2: second HA elution; MW: molecular weights (in KDa); putative CDK955 and CDK942 isoforms are indicated by arrows; compared to the Figure 1A, linear adjustment of contrast and brightness was applied by Photoshop software to the whole image; D) Protein expression analysis. Western blot assays of total cell lysates were performed with non-target (Nt-sh) or Ikaros-specific (Ik-sh) sh-RNA Jurkat clones; fold decrease of IKAROS protein levels [file pgen.1004827.s001.tif]

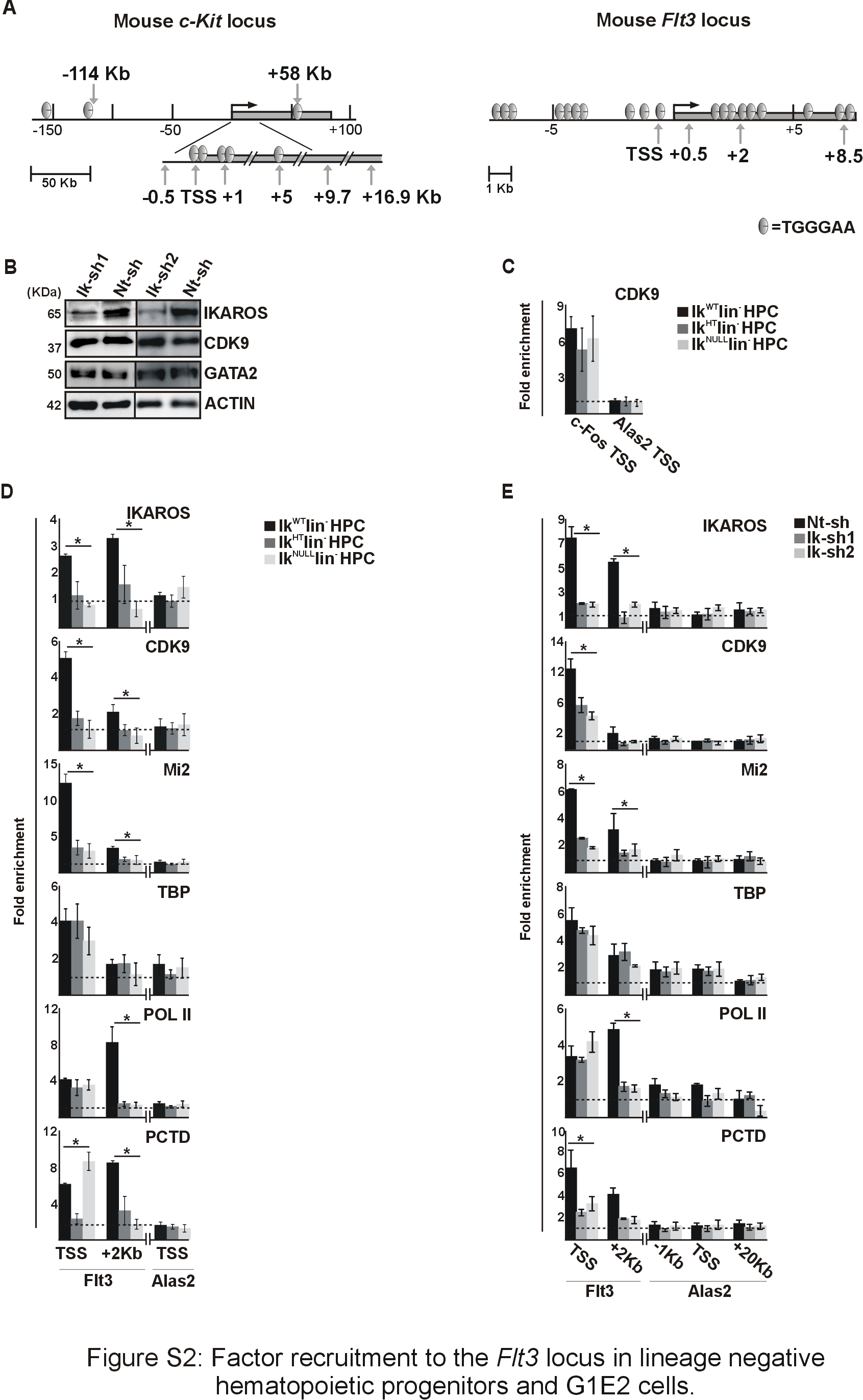

Supplement: Figure S2 — Factor recruitment to the Flt3 locus in lineage negative hematopoietic progenitors and G1E2 cells. A) Schematic representation of the murine c-Kit and Flt3 loci. Amplicon positions are indicated with arrows [44], [81]. For the c-Kit locus, they correspond to: −114 Kb enhancer region; −0.5 Kb region; the Transcriptional Start Site (TSS); +1 Kb, +5 Kb, +9.7 Kb, +16.9 Kb and +58 Kb Open Reading Frame (ORF) regions; for the Flt3 locus, the TSS and the ORF +0.5 Kb, +2 Kb and +8.5 Kb regions; filled circles: TGGGAA IKAROS consensus binding sites that IKAROS specifically and directly binds in crude nuclear extracts of hematopietic cells [7], [79]; B) Protein expression analysis. Western blot assays of total cell lysates of Ikaros knock-down G1E2 clones; ACTIN was used as internal control; Nt-sh: non-target sh-RNA G1E2 clones; Ik-sh: Ikaros-specific sh-RNA (Ik-sh1 and Ik-sh2) G1E2 clones; C–E) Chromatin immunoprecipitation (ChIP). ChIP assays were carried out with the antibodies indicated on the top of each panel; POL II: is an antibody against the N-terminal region of the large subunit of POL II and binds POL II in a phosphorylation-independent manner; PCTD: is an antibody against the CTD repeats phosphorylated at Ser2; y-axis: fold enrichments of Flt3 or Alas2 regions relative to Thp promoter and input samples are plotted as the mean ± Standard Deviation (SD) of the measurements; a value of 1 (dotted lines) indicates no enrichment; n≥4; the amplicons tested recognized the c-Fos TSS, the Flt3 TSS and ORF +2 Kb regions as well as the Alas2 −1 Kb, TSS and +20 Kb ORF regions. Since the Alas2 gene is not expressed in G1E2 cells, it was used as negative control; the Alas2 values shown in panel C correspond to the Alas2 values displayed in Figure 2D (CDK9 ChIP); lin−: bone marrow-derived lineage negative hematopoietic progenitor cells (HPCs); IkWT lin−: Ikaros wild type lin− HPCs; IkHT: Ikaros heterozygote null lin− HPCs; IkNULL lin−: Ikaros homozygote null lin− HPCs; Nt-sh: non [file pgen.1004827.s002.tif]

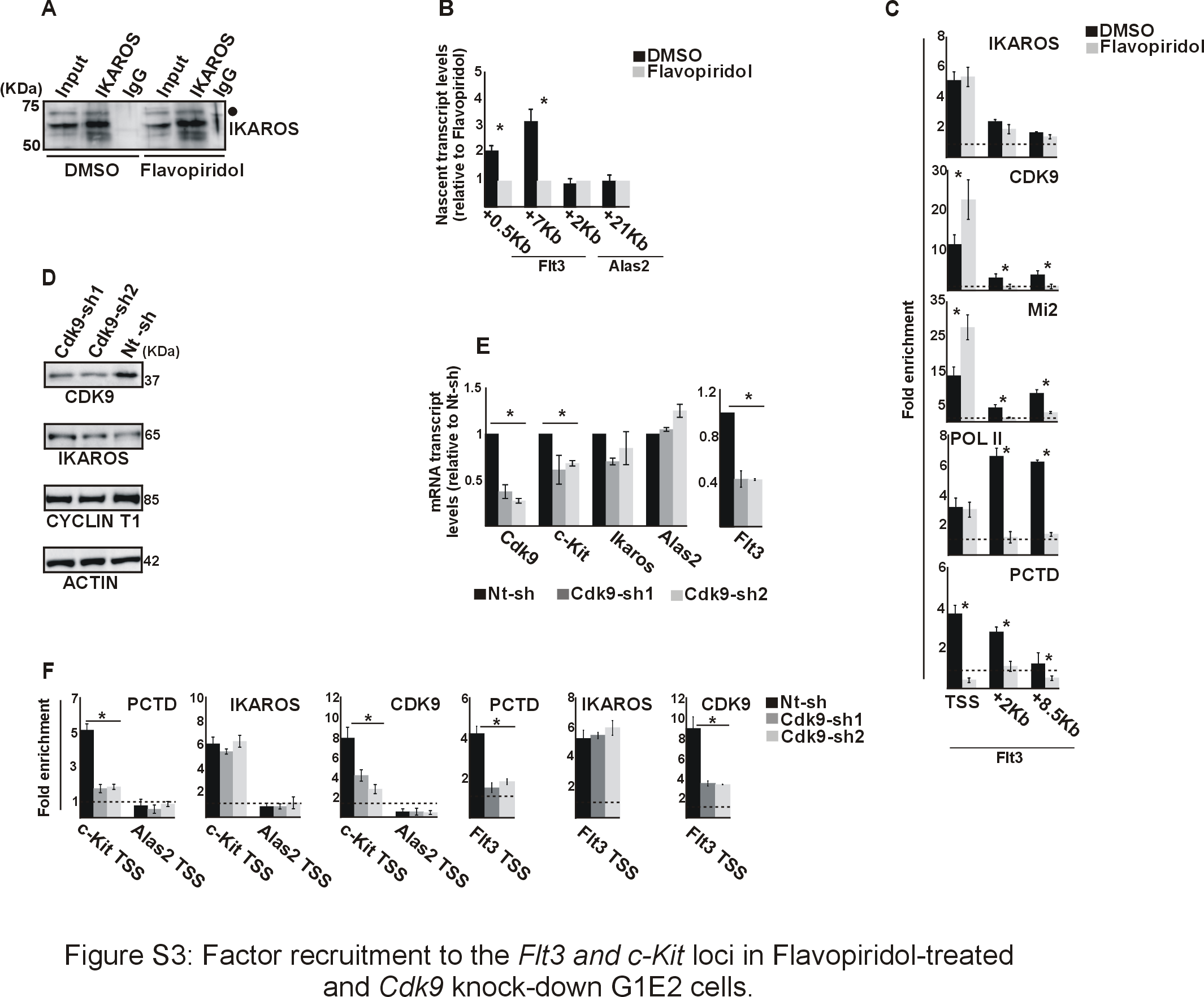

Supplement: Figure S3 — Factor recruitment to the Flt3 and c-Kit loci in Flavopiridol-treated and Cdk9 knock-down G1E2 cells. In panels A–C, G1E2 cells were treated for 2 h either with 0.01% DMSO (control) or 100 nM Flavopiridol. A) Protein co-immunoprecipitation of G1E2 total cell lysates. Immunoprecipitations were performed with an IKAROS-specific antibody and immunoblots were probed with the same antibody in order to exclude any significant variation of IKAROS phosphorylation levels upon Flavopiridol treatment; Input samples represent 2% of total cell lysates; filled dot: non-specific band; B) Gene expression profiles. RNA samples were retro-transcribed with random oligonucleotides to amplify nascent transcripts, which were used as template for qPCR with intron-specific Flt3 (+0.5 Kb, +7 Kb regions) or Alas2 (+2 Kb, +21 Kb regions, used as negative control) primer sets; Rnu2-1, a Flavopiridol-insensitive gene was used as internal control; y axis: relative nascent transcript enrichment levels; ratios are plotted as the mean ± Standard Deviation (SD) of the measurements; n≥4; C, F) Chromatin immunoprecipitation (ChIP). ChIP assays were carried out with the antibodies labeled on the top of each panel; POL II: is an antibody against the N-terminal region of the large subunit of POL II and binds POL II in a phosphorylation-independent manner; PCTD: is an antibody against the CTD repeats phosphorylated at Ser2; y-axis: fold enrichments of Flt3 TSS or +2 and +8.5 ORF regions, c-Kit and Alas2 TSS, relative to Thp promoter and input samples are plotted as the mean ± SD of the measurements; a value of 1 (dotted lines) indicates no enrichment; n≥4; D) Protein expression analysis. Western blot assays of total cell lysates of non-target (Nt-sh) or Cdk9-specific (Cdk9-sh1 and Cdk9-sh2) sh-RNA G1E2 clones; the antibodies used are indicated at the bottom of the panels; ACTIN was used as internal control; E) Gene expression profiles. mRNA samples were retro-transcribed with oligo-dT nucleotides; cDNA wa [file pgen.1004827.s003.tif]

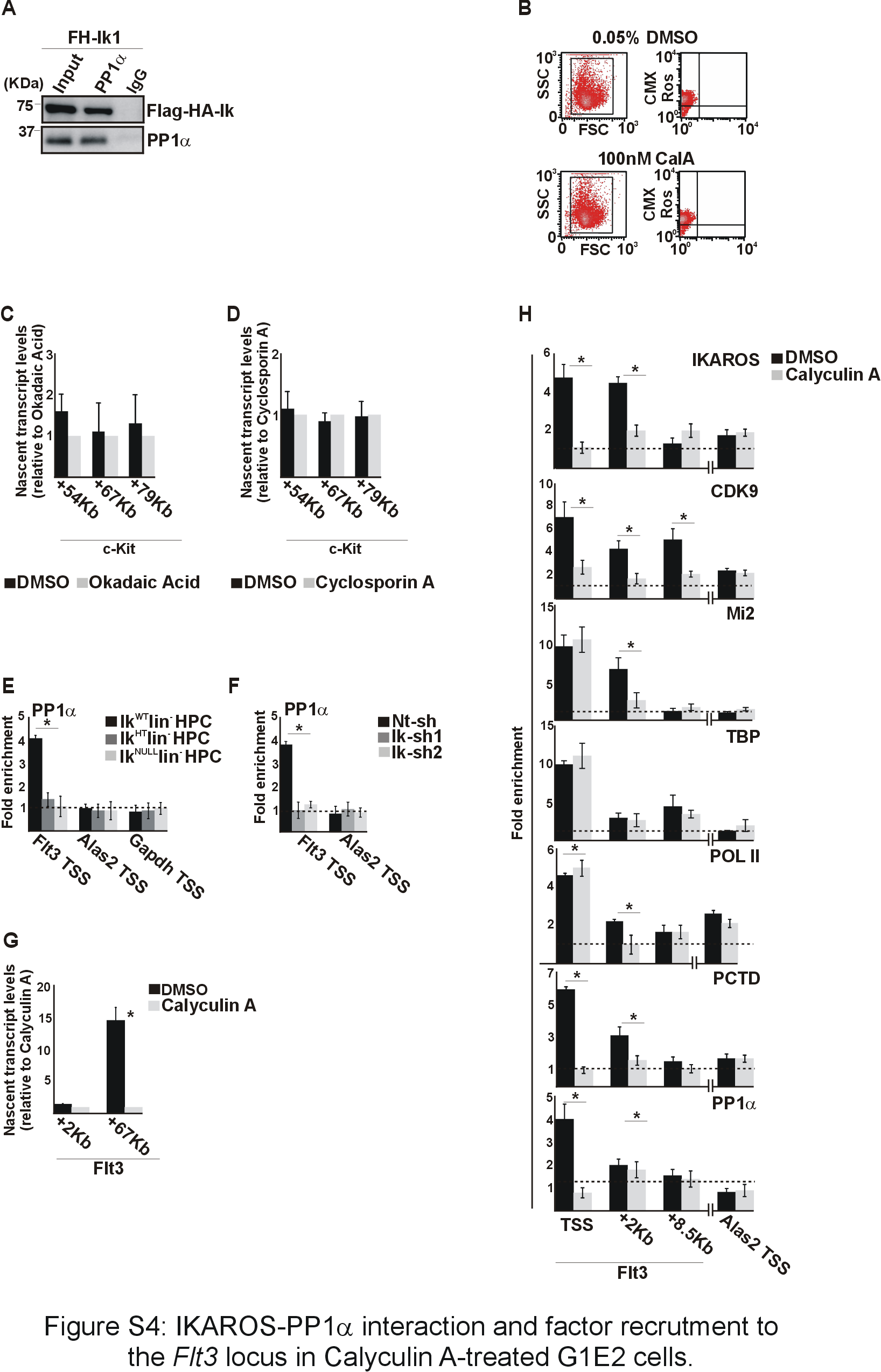

Supplement: Figure S4 — IKAROS-PP1α interaction and factor recruitment to the Flt3 locus in Calyculin A-treated G1E2 cells. A) Protein co-immunoprecipitation of COS-7 total cell lysates. COS-7 cells, which do not express any IKAROS protein, were transiently transfected with an expression vector encoding the murine Ikaros1 cDNA with a Flag and HA tag at its N-terminal region (FH-Ik1); immunoprecipitations were performed with PP1α antibodies or isotype-matched IgG control (IgG); immunoblots were probed with antibodies directed against HA (for Flag-HA-Ik detection; upper panel) or PP1α (lower panel); Input samples represent 2% of nuclear extracts; B) CMX Ros staining of DMSO- (upper panels) or Calyculin A- (CalA, lower panels) treated G1E2 cells. Flow cytometry; FCS: Forward scatter; SSC: Side scatter; CMX Ros: red fluorescence emission from CMX Ros; C, D, G) Gene expression profiles of G1E2 cells. RNA samples were retro-transcribed with random oligonucleotides to amplify nascent transcripts, which were used as templates for qPCR with intron-specific c-Kit (+54 Kb, +67 Kb and +79 Kb regions), Flt3 (+2 Kb and +67 Kb regions) or Gapdh (used as internal control) primer sets; y axis: relative nascent transcript enrichment levels; ratios are plotted as the mean ± Standard Deviations (SD) of the measurements; n≥4; in panel C, the experiments were performed on G1E2 cells treated for 30 min with 1 µM Okadaic Acid or its diluent as control; in panel D, the experiments were performed on G1E2 cells treated for 2 h with 1 µM Cyclosporin A or its diluent as control; in panel G, the experiments were performed on G1E2 cells treated for 30 min with 100 nM Calyculin A or its diluent as control; E, F, H) Chromatin Immunoprecipitation (ChIP). ChIP assays were carried out with the antibodies labeled on the top of each panel; POL II: is an antibody against the N-terminal region of the large subunit of POL II and binds POL II in a phosphorylation-independent manner; PCTD: is an antibody against the CTD repeats pho [file pgen.1004827.s004.tif]

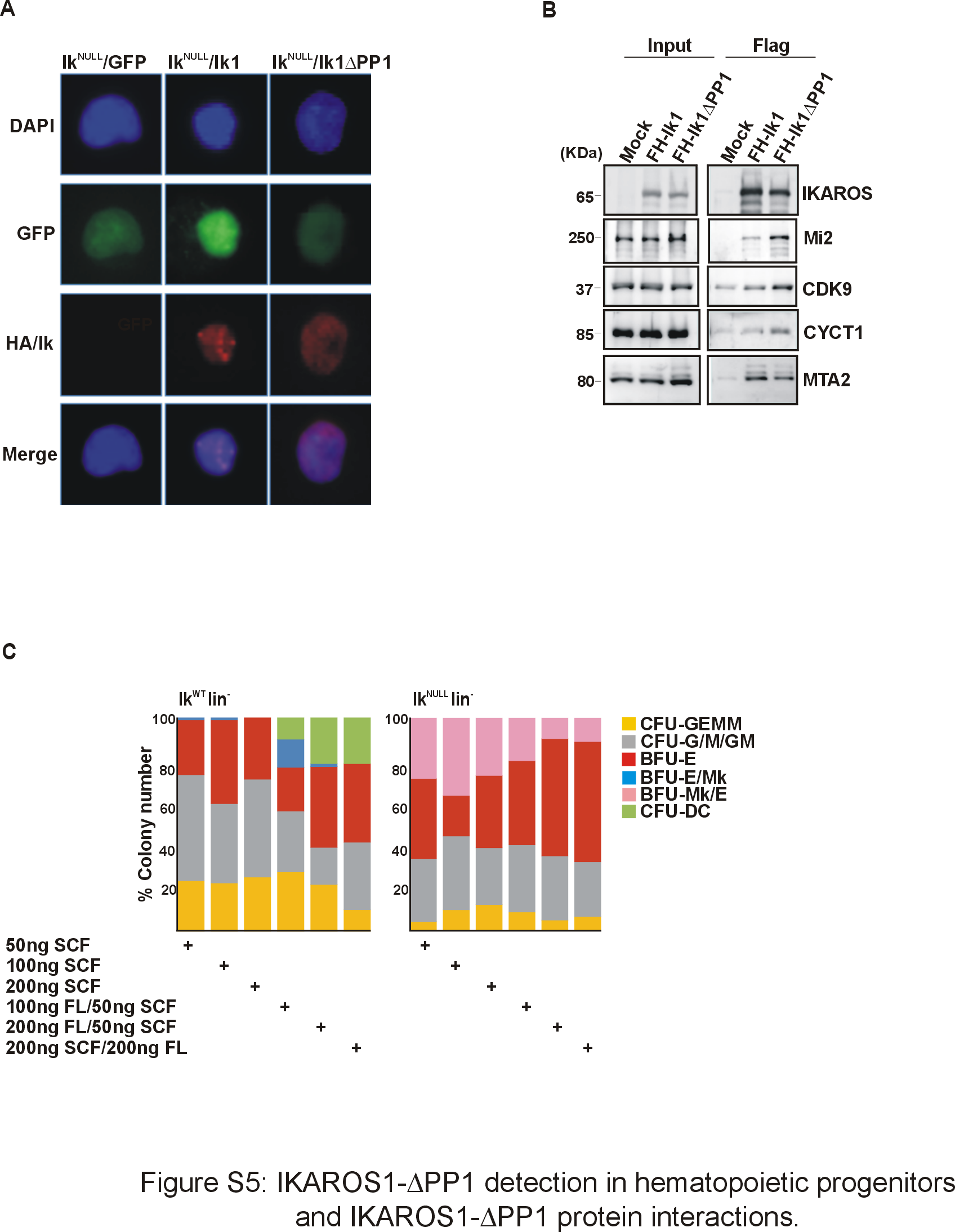

Supplement: Figure S5 — IKAROS1-ΔPP1 detection in hematopoietic progenitors and IKAROS1-ΔPP1 protein interactions. A) Single-cell immunofluorescence analysis. Ikaros homozygote null bone marrow-derived lineage negative hematopoietic progenitor cells (IkNULL lin− HPCs) were transduced with pMSCV/Ik1 (IkNULL/Ik1), pMSCV/Ik1ΔPP1 (IkNULL/Ik1ΔPP1) or the empty pMSCV vector (IkNULL/GFP) and then cytospun and fixed on slides; infected cells were recognized as GFP+ cells; IKAROS1 or IKAROS1ΔPP1 proteins were detected with mouse anti-HA and Texas Red (TR)-conjugated anti-mouse antibody; representative lin− HPCs transduced cells are shown where infected cells are detected as green signals and IKAROS1- or IKAROS1ΔPP1-expressing cells are detected as red signals; B) Protein co-immunoprecipitation of 293T total cell lysates. 293T cells, which do not express any IKAROS protein, were transiently transfected with an expression vector encoding the murine Ikaros1 cDNA (FH-Ik1), the mutant Ikaros1ΔPP1 (FH-Ik1ΔPP1) cDNA or the empty vector (Mock), all with a Flag and HA tag at their N-terminal regions; immunoprecipitations were performed with FLAG antibodies; immunoblots were probed with IKAROS, Mi2, CDK9, CYCLIN T1 or MTA2 antibody; Input samples represent 2% of total cell lysates; C) In vitro hematopoietic differentiation of lineage negative (lin−) hematopoietic progenitor cells (HPCs). Clonogenic assays in methylcellulose of IkWT or IkNULL lin− HPCs grown for 3 days in cytokine-supplemented liquid cultures; lin− HPCs were seeded on methylcellulose and colonies were scored at day 14; CFU-GEMM: colony forming unit granulocyte, erythrocyte, macrophage, megakaryocyte; CFU-G/M/GM: collectively identifies granulo-macrophage colonies; BFU-E: burst-forming unit erythrocyte; BFU-E/Mk: immature erythroid colonies with elevated megakaryocytic content; BFU-Mk/E: almost pure megakaryocyte colonies that contain only few clusters of immature erythroid cells; CFU-DC: colony forming unit dendritic cell; the data shown are [file pgen.1004827.s005.tif]
