# Supplementary material for: The IKAROS Interaction with a Complex Including Chromatin Remodeling and Transcription Elongation Activities Is Required for Hematopoiesis
Source: PLoS Genet. 2014 Dec 4;10(12):e1004827. doi: 10.1371/journal.pgen.1004827 (PMC4256266; doi:10.1371/journal.pgen.1004827)
Supplement: Table S1 — Flt3 POL II traveling ratio. Flt3 traveling ratio values (as defined by the relative ratio of POL II density in gene ORF vs. promoter-proximal regions) were obtained by chromatin immunoprecipitation with POL II antibody, which recognizes the N-terminal region of the large subunit of POL II and binds POL II in a phosphorylation-independent manner and indicate the enrichment levels of Flt3 +2/TSS regions relative to the control and the input samples (see also Figure S2 and S3 legends); IkWT: Ikaros wild type HPCs; IkHT: Ikaros heterozygote null HPCs; IkNULL: Ikaros homozygote null lin− HPCs; DMSO: Dimethyl sulfoxide-treated G1E2 cells (0.01% for 2 h); Fvp: Flavopiridol-treated G1E2 cells (100 nM for 2 h). (DOCX) [file pgen.1004827.s006.docx]

**Table S1. *Flt3* POL II Traveling Ratio.**

|  | **Ik^WT^** | **Ik^HT^** | **Ik^NULL^** | **DMSO** | **Fvp** |
| --- | --- | --- | --- | --- | --- |
| *Flt3 TR* | 1.98 | 0.45 | 0.39 | 2.06 | 0.39 |
