# Supplementary material for: The IKAROS Interaction with a Complex Including Chromatin Remodeling and Transcription Elongation Activities Is Required for Hematopoiesis
Source: PLoS Genet. 2014 Dec 4;10(12):e1004827. doi: 10.1371/journal.pgen.1004827 (PMC4256266; doi:10.1371/journal.pgen.1004827)
Supplement: Table S2 — Protein phosphatase 1 catalytic subunits identified by immunoaffinity purification and LC-MS/MS analysis of Flag-HA-IKAROS complexes. The percentage values indicate the sequence coverage of the identified proteins; False Discovery Rate (FDR): 0%. (DOCX) [file pgen.1004827.s007.docx]

**Table S2. Protein phosphatase 1 catalytic subunits identified by immunoaffinity purification and LC-MS/MS analysis of Flag-HA-IKAROS complexes.**

| **Unique Peptides** | **Total Peptides** | **Reference** | **Gene Symbol** | **AVG** | **Coverage** |
| --- | --- | --- | --- | --- | --- |
| **8** | **14** | **PP1A_HUMAN** | **PPP1CA (PP1α)** | **2.7986** | **32,73%** |
| 2 | 2 | PP1B_HUMAN | PPP1CB (PP1β) | 2.5512 | 3,06% |
| 1 | 1 | PP1G_HUMAN | PPP1CC (PP1γ) | 2.8515 | 3,10% |
