# Supplementary material for: The IKAROS Interaction with a Complex Including Chromatin Remodeling and Transcription Elongation Activities Is Required for Hematopoiesis
Source: PLoS Genet. 2014 Dec 4;10(12):e1004827. doi: 10.1371/journal.pgen.1004827 (PMC4256266; doi:10.1371/journal.pgen.1004827)
Supplement: Table S3 — Super Elongation Complex (SEC) and microprocessor components identified by immunoaffinity purification and LC-MS/MS analysis of Flag-HA-IKAROS complexes. False Discovery Rate (FDR): 0%. (DOCX) [file pgen.1004827.s008.docx]

**Table S3. Super Elongation Complex (SEC) and Microprocessor components identified by immunoaffinity purification and LC-MS/MS analysis of Flag-HA-IKAROS complexes.**

| **Unique Peptides** | **Total Peptides** | **Reference** | **Gene Symbol** | **AVG** | **Complex** |
| --- | --- | --- | --- | --- | --- |
| 22 | 28 | HCFC1_HUMAN | HCFC1 | 3.050 | SEC |
| 7 | 7 | SET1A_HUMAN | SETD1A | 2.3149 | SEC |
| 7 | 9 | WDR82_HUMAN | WDR82 | 3.4390 | SEC |
| 6 | 6 | MLL1_HUMAN | MLL | 2.5747 | SEC |
| 6 | 9 | WDR5_HUMAN | WDR5 | 3.1764 | SEC |
| 3 | 4 | ASH2L_HUMAN | ASH2L | 3.0562 | SEC |
| 3 | 3 | RBBP5_HUMAN | RBBP5 | 2.9745 | SEC |
| 2 | 2 | WDR18_HUMAN | WDR18 | 2.1065 | SEC |
| 1 | 1 | CGBP1_HUMAN | CGGBP1 | 2.4261 | SEC |
|  |  |  |  |  |  |
| 11 | 12 | XRN2_HUMAN | XRN2 | 3.4120 | Microprocessor |
| 5 | 5 | SETX_HUMAN | SETX | 2.4013 | Microprocessor |
| 23 | 26 | SYMPK_HUMAN | SYMPK | 3.0454 | Polyadenylation machinery |
