# Supplementary material for: The IKAROS Interaction with a Complex Including Chromatin Remodeling and Transcription Elongation Activities Is Required for Hematopoiesis
Source: PLoS Genet. 2014 Dec 4;10(12):e1004827. doi: 10.1371/journal.pgen.1004827 (PMC4256266; doi:10.1371/journal.pgen.1004827)
Supplement: Text S1 — Supplemental methods. (DOCX) [file pgen.1004827.s009.docx]

**SUPPLEMENTAL METHODS**

**Antibodies**

## Mi2 (H-242), IKAROS (H-100), CDK9 (C-20), CYCT1 (H-245), PP1α (N-19), PP1 (E-9), POL II (N-20), HA (Hemagglutinin) (Y-11; F7), BRD4 (H-250), TBP (SI-1), MTA2 (C-20), MBD3 (C-18), GATA2 (CG2-96), HEXIM1 (Q-20), PARP-1 (F-2) antibodies were purchased from Santa Cruz Biotechnology; APE1 (MAB1044) antibody from R&D Systems; ACTIN (4968) antibody from Cell Signaling; Mi2 (ab70469), HISTONE H3 (ab1791), MBD3 (ab3755), PCNA (PC10) antibodies from Abcam; RNA POL II phoshpo-CTD antibodies were purchased from Chromotek (clone 3E10) or Covance (clone H5); RBBP4 antibody (mouse monoclonal; clone 15G12) was a generous gift of Dr A. Verreault, IRIC). The HRP-conjugated light chain-specific secondary antibodies used in Western blot assays of co-immunoprecipitation experiments were the following: Goat TrueBlot®: Anti-Goat IgG HRP; 18-8814-31 (monoclonal, eB270) (from Rockland); Mouse TrueBlot® ULTRA: Anti-Mouse Ig HRP; 18-8817-31 (monoclonal, eB144) (from Rockland) and Peroxidase IgG Fraction Monoclonal Mouse Anti-Rabbit IgG, Light Chain Specific; 211-032-171 (whole IgG affinity-purified antibodies) (from Jackson ImmunoResearch). Anti-Flag M2 agarose affinity gel and anti-Hemagglutinin (HA) resin from Sigma-Aldrich; mouse, goat, sheep isotype-matched immunoglobulins were purchased from SantaCruz Biotechnology; rabbit isotype-matched immunoglobulins from Millipore. Antibodies and isotype-matched immunoglobulins were used for protein co-immunoprecipitation, western blot analysis and/or chromatin immunoprecipitation assays.

**Reagents**

Calyculin A (30 min, 100 nM), Okadaic Acid (30 min, 10 nM) and Cyclosporin A (2 h, 1 μM) were purchased from Santa Cruz Biotechnology; Flavopiridol (2 h, 100 nM) from Sigma-Aldrich.

Murine *Ikaros*-specific sh-RNAs were purchased from OriGene Technologies Inc. (pGFP-V-RS; GI561140) and Sigma-Aldrich (pLKO.1-puro; TRCN85513). *Cdk9*-specific sh-RNAs were purchased from OriGene Technologies Inc. (pGFP-V-RS; GI592566 and GI592568). *Mi2*-specific sh-RNAs were purchased from OriGene Technologies Inc. (pGFP-V-RS; GI590722 and GI590723). *Human* Ikaros-specific sh-RNAs were purchased from Sigma-Aldrich (pLKO.1-puro; TRCN107872).

**Protein co-immunoprecipitation.**

Protein co-immunoprecipitation in COS-7 and 293T cells were done essentially as previously reported[[27](#_ENREF_1)]. Protein co-immunoprecipitation in Jurkat and G1E2 cells were conducted on 3-5 mg of nuclear proteins. Nuclear extracts were incubated with 2 μg of specific antibodies for 8 h at 4°C in immunoprecipitation buffer (50 mM Tris [pH 7.3], 150 mM NaCl, 5 mM EDTA, 10 mM NaF, 1% Triton X-100) with 1 mM PMSF and protease inhibitors (Sigma-Aldrich). Immunocomplexes were isolated with protein G-Sepharose beads saturated with 1% BSA, by gentle rocking for 4 h at 4°C. Beads were washed 5-8 times with ice-cold IP buffer. Bound proteins were retrieved from Sepharose beads by boiling in Laemmli buffer containing β-mercaptoethanol.

**Fast Protein Liquid Chromatography (FPLC).**

Protein complexes were purified on an AKTA Purifier FPLC System (GE Healthcare) using a SuperoseTM 6 10/300GL column (GE Healthcare). This column has an exclusion limit of 4X10^7^ Dalton (Da) and an optimal separation range of 5000-5X10^6^ Da. Column equilibration and size fractionation was carried out in elution buffer (50 mM Tris-HCl [pH 7.3], 10% glycerol, 60 mM KCl, 0.2 mM EDTA, 0.1% NP-40, 0.5 mM DTT). The molecular masses of the 500 μl-eluted fractions were estimated by column standardization with protein standards (GE Healthcare). Each column fraction was precipitated with 15% trichloroacetic acid, centrifuged at 20000 g for 20 min, washed once in ice-cold acetone, resuspended in loading buffer, loaded onto 10% SDS-polyacrylamide gels and electroblotted onto nitrocellulose membranes.

**Quantitative sequential ChIP (re-ChIP) analysis.**

Quantitative re-ChIP analysis was carried out with the Percent Input Method.

**Cell culture, transient and stable cell transfections.**

The GIE2 cell line is a GATA1 knock-out proliferating progenitor cell line. The derived G1E-ER4 cells express a *Gata1*-estrogen receptor transgene and upon estradiol treatment they undergo synchronous differentiation recapitulating early stages of erythroid differentiation[S1,S2]. G1E2 cells were maintained in culture as previously described[[4](#_ENREF_4)3]. The other cell lines were grown in DMEM (COS-7 and 293T cells) or RPMI (Jurkat cells) medium containing 1% penicillin/streptomycin and 10% fetal bovine serum and maintained at 37°C and 5% CO2. To generate G1E2 knock-down, 3 million cells were resuspended in 100 μl of Nucleofector Solution R and transfected with 10 μg of linearized and endotoxin-free Maxi-prep DNA (Qiagen) using the G-016 program with the Nucleofector II system (Amaxa Biosystems). *Ikaros* as well as *Cdk9* knock-down cells were selected with Puromycin (2 μg/ml; InvivoGen) and screened by Western blot for target protein knock-down. Stable *Mi2* knock-down cells were not viable. Therefore, *Mi2* knock-down cells were isolated based on their GFP fluorescence 48 hr after nucleoporation and used to prepare primary as well as mature transcripts. The level of *Mi2* knock-down was calculated at the transcriptional level.

Ik1 and Ik2 are full-length and functional IKAROS isoforms[[78-80](#_ENREF_5)]. Ik1 has been inserted into expression vectors used to generate COS-7 and 293T cell transient transfected cells as well as primary bone marrow-derived lineage negative (lin^-^) hematopoietic progenitor cells (HPCs). Ik2 has been inserted into the expression vector used to generate Jurkat cell lines. To generate Jurkat cells expressing IKAROS, cells were infected with pOZ-N-Flag-HA-IRES-ILR2 (mock sample) or pOZ-N-Flag-HA-IKAROS-IRES-ILR2- (Flag-HA-Ik sample) vector[[76](#_ENREF_8)]. Recombinant clones were purified using anti-IL2 magnetic beads (Life Technology)[[76](#_ENREF_8)] and screened by western blot with HA or Flag specific antibodies. To generate Jurkat cells knocked-down for *Ikaros*, cells were infected with non-target or *Ikaros* knock-down vector. Positive clones were selected with Puromycin (2 μg/ml; InvivoGen) and screened by Western blot for *Ikaros* knock-down. COS-7 and 293T cells were transiently transfected with expression vectors encoding the murine *Ikaros1* cDNA or the mutant *Ikaros1ΔPP1* cDNA both with a Flag and HA tags at their N-terminal regions (FH-Ik1 or FH-Ik1ΔPP1). COS-7 and 293T cells were transfected with FuGene HD reagent (Promega) or PEI according to manufacturer’s instructions, and harvested after 48 hours.

**Sequential immunoaffinity purification of IKAROS-associated proteins.**

Flag-HA sequential immunoaffinity (or Tap-Tag) purification was carried out as described in Nakatani et *al.*[[76](#_ENREF_8)] starting from nuclear extracts prepared from pOZ-N-Flag-HA-IRES-ILR2 (mock sample) or pOZ-N-Flag-HA-IKAROS-IRES-ILR2 (Flag-HA-Ik sample) Jurkat cell clones. The extracts were first clarified by centrifugation at 30000 g for 1 h, followed by filtration through a 0.45 μm pore filter. The lysates were then incubated with the anti-Flag M2 resin (Sigma-Aldrich) overnight and then, extensively washed with immunoprecipitation buffer (50 mM Tris [pH 7.3], 150 mM NaCl, 5 mM EDTA, 10 mM NaF, 1% Triton X-100) with 1 mM PMSF and protease inhibitors (Sigma-Aldrich). The resin was eluted three times with 200 μg/ml of Flag peptide (Sigma-Aldrich). The eluted fractions were incubated with the anti-HA resin (Sigma-Aldrich) overnight. The following day the resin was extensively washed and protein immune-complexes were eluted with 200 μg/ml of HA peptides (Sigma-Aldrich). One sixtieth of each of the HA eluted fractions was used for silver staining (Invitrogen) according to manufacturer’s instructions. Then, the first and second elution fractions were pooled, protein were precipitated with 15% trichloroacetic acid, centrifuged at 20000 g for 20 min, washed once with ice-cold acetone, resuspended in Laemmli buffer and loaded onto pre-cast NUPAGE 4-12% Bis Tris gel (Invitrogen). SDS-PAGE was stained with Sypro-Ruby (Invitrogen). Excised bands underwent LC-MS/MS analysis at the Taplin Mass Spectrometry Facility (Harvard University, Boston).

**Retrovirus infection.**

VSV cells were transfected with Lipofectamine 2000 (Invitrogen). The resulting retroviral supernatant was collected, filtered through a 0.2 μm low protein binding filter (Millipore), supplemented with 6 μg/ml of polybrene (Millipore) and used to infect GP+E-86 cells. Three rounds of infection were needed to obtain 100% of transduced cells. Then GP+E-86 viral supernatant was collected, filtered, supplemented with 4 μg/ml of polybrene and used to infect lin^-^ HPCs.

**Immunofluorescence analysis.**

Single-cell immunofluorescence analysis was performed as reported in Bottardi et *al*.[[23](#_ENREF_9)].

**Primer sets for cDNA.**

Primer name 5’ Primer sequence 3’ Primer sequence

*c-Kit* CAAATTCACCCTCAAAGTGCG GCCACATGGAGTTCACGGAT

*Flt3* CGCCACAGGCCCAGGTGAAG TCCAGCGAAGCAACGGCCTG

*Mi2* AGGGTGAAATGAATCGTGGC CGAAGCTGCTCCTCGATCAC

*Gata2* GAGCTCCGTGGCCTCCCTCA CCCCGGGCTACACTACCCCC

*Cdk9* CTCCGGAGGCCGGTACCACA GATCTGGCTGCCCTTCCGCC

*βActin* ATCGTGGGCCGCCCTAGGCACCA TCCATGTCGTCCCAGTTGGTAACAA

*Ikaros* AATGCCATCAACTACCTGGG CAGCAGCAGCAAGTTATCCA

*Alas2* GCAGCAGCTATGTTGCTACG ACAGGTTGGTCCTTGAGTGG

**Primer sets for nascent transcripts and Chromatin Immunoprecipitation assays.**

Primer name 5’ Primer sequence 3’ Primer sequence

Thp-pr GGTGGATGGTGTGGTCACAAC GGTCTTGACACACCAGCTTT

Gapdh-pr CAGCTACTCGCGGCTTTAC CTGGCACTGCACAAGAAGA

c-Fos pr CTGTTCCCGTCAATCCCTCC TCCTAGACCTTCCGCGTGTA

Gapdh prtrx TGGAGGAGGTTTGCTGGGCAAG GCCTGGCAGGGCTTTTATAGCACG

Rnu2-1 prtrx TCGCTTCTCGGCCTTTTGGCT TGCGTGGAGTGGACGGAGCA

c-Kit +1 GACGCCTGGGACAACCAGCC TGCACAAAGAGCGCCCGAGG

c-Kit TSS GCGAGAGCTGTAGCAGAGAG CAACAGGACGCAGAGCAGAT

c-Kit +49Kb GGGGCGGGGTTCGGTTCTTG TCTGACCGCACCCAAACGGC

c-Kit +54Kb TTGGTCCAGGAGGGCGTCGT CCAGCATCCGTGGAAGCCCC

c-Kit +67Kb AGAGTGCCACCAAGGGTGCG ATGGTGTGCAGAGTGCCGGG

c-Kit +77Kb GGTGCGTGAGGGGTGCACTT GACGGCTGGCTGTTGGTCCG

c-Kit +79Kb GGGATGCCCGCAGCTGTGTAG GGAGGGGGAGGGCGGCTTAAA

Flt3 TSS ACCTCTCTCCAGAGGCCGCG GCCGAACCGCACCCAGTCTC

Flt3 +0.5Kb CCCCTGCCCTCACCCTCCTCC GCCACCTGCGCTGCTGAAGAAGA

Flt3 +2Kb CCAAGGCAACCAATAACAACG AGACTCCAGGCCTCACGTACA

Flt3 +8.5Kb ATAGCTGGCACCTCAGCCGC CCAGGGCTGTGCTGAGCCTC

Flt3 +67Kb AGGGTGGGTATGGATGGGAA AGCAGACCTCTCCTGTGCTA

Alas2 -1Kb GAGTGTGGGTTCTGGGAGCA ACTGGGAGGGTGGGCTACTAA

Alas2 TSS AGCAGGAGGCTGGGATGAA TCTTACCTGTTGCCCTGCACT

Alas2 +20Kb GGCCTGCCACAGGCGTTTTG CAAGCACAGTGCCCAGCCCA

The following primer sets for the murine *c-Kit* locus were synthesized according to Jing et *al*.[[44](#_ENREF_10)]: 114 (8F+8R); 0 (-0.5 region throughout the text) (20F+20R); +4.7 (+5 region throughout the text) (21F+21R); +9.7 (22F+22R); +16.9 (24F+24R); +58.1 (32F+32R).

**SUPPLEMENTAL REFERENCES**

S1. Cheng Y, Wu W, Kumar SA, Yu D, Deng W, et al. (2009) Erythroid GATA1 function revealed by genome-wide analysis of transcription factor occupancy, histone modifications, and mRNA expression. Genome Res 19: 2172-2184.

S2. Welch JJ, Watts JA, Vakoc CR, Yao Y, Wang H, et al. (2004) Global regulation of erythroid gene expression by transcription factor GATA-1. Blood 104: 3136-3147.
